# A multi-modal dataset for insect biodiversity with imagery and DNA at the trap and individual level

**DOI:** 10.1038/s41597-026-07251-x

**Published:** 2026-04-21

**Authors:** Johanna Orsholm, John Quinto, Hannu Autto, Gaia Banelyte, Nicolas Chazot, Jeremy deWaard, Stephanie deWaard, Arielle Farrell, Brendan Furneaux, Bess Hardwick, Nao Ito, Amlan Kar, Oula Kalttopää, Deirdre Kerdraon, Erik Kristensen, Jaclyn McKeown, Tommi Mononen, Ellen Nein, Hanna Rogers, Tomas Roslin, Paula Schmitz, Jayme Sones, Maija Sujala, Amy Thompson, Evgeny V. Zakharov, Iuliia Zarubiieva, Akshita Gupta, Scott C. Lowe, Graham W. Taylor

**Affiliations:** 1 Department of Ecology, Swedish University of Agricultural Sciences, Uppsala, Sweden; 2 University of Guelph, Guelph, Ontario Canada; 3 Vector Institute, Toronto, Ontario Canada; 4 Kilpisjärvi Biological Station, University of Helsinki, Helsinki, Finland; 5 Centre for Biodiversity Genomics, University of Guelph, Guelph, Ontario Canada; 6 Department of Entomology, National Museum of Natural History, Smithsonian Institution, Washington, US; 7 Department of Biological and Environmental Science, University of Jyväskylä, Jyväskylä, Finland; 8 Faculty of Biological and Environmental Sciences, University of Helsinki, Helsinki, Finland; 9 NVIDIA, Toronto, Ontario Canada; 10 University of Toronto, Toronto, Ontario Canada; 11 Unit for Field-based Forest Research, Swedish University of Agricultural Sciences, Umeå, Sweden; 12 TU Darmstadt, Darmstadt, Germany

**Keywords:** Machine learning, High-throughput screening, Image processing

## Abstract

Insects comprise millions of species, many experiencing severe population declines under environmental and habitat changes. High-throughput approaches are crucial for accelerating our understanding of insect diversity, with DNA barcoding and high-resolution imaging showing strong potential for automatic taxonomic classification. However, most image-based approaches rely on individual specimen data, unlike the unsorted bulk samples collected in large-scale ecological surveys. We present the Mixed Arthropod Sample Segmentation and Identification (MassID45) dataset for training automatic classifiers of bulk insect samples. It uniquely combines molecular and imaging data at both the unsorted sample level and the full set of individual specimens. Human annotators, supported by an AI-assisted tool, performed two tasks on bulk images: creating segmentation masks around each individual arthropod and assigning taxonomic labels to over 17000 specimens. Combining the taxonomic resolution of DNA barcodes with precise abundance estimates of bulk images holds great potential for rapid, large-scale characterization of insect communities. This dataset pushes the boundaries of tiny object detection and instance segmentation, fostering innovation in both ecological and machine learning research.

## Background & Summary

Insects are the most diverse organism group on Earth, with more than one million described species^1^ and an estimated four million species yet to be discovered^2^. Climate change and anthropogenic activities are causing rapid declines in insect populations^3^, and many species likely face extinction in the coming decades^4^. Yet our understanding of insect diversity is seriously data-limited and incomplete. In particular, data generation is hampered by a severe lack of available taxonomic expertise^5,6^. Consequently, there is an urgent need for high-throughput methods to study and monitor insect communities, with machine learning-based image classification and molecular methods quickly advancing as promising tools for the taxonomic characterization of samples.

To taxonomically classify diverse insect samples, substantial amounts of training data are needed. This requires taxonomically labelled examples — images for visual classification and DNA sequences for molecular approaches. At present, the most abundant insect DNA sequence data consists of short fragments (typically 658 bp) from a standardized gene region, the cytochrome c oxidase subunit 1 (COI), commonly known as DNA barcodes^7^. Comprehensive datasets containing millions of images and DNA barcodes from individual insect specimens are now available for training classifiers^8–10^. However, many large-scale ecological studies or monitoring efforts collect insects in bulk samples, which contain multiple specimens with mixed taxonomic composition. Sorting bulk samples into individual specimens requires substantial effort. Thus, for many ecological applications, there is currently a discrepancy between the available specimen-level training data and the bulk-level samples in need of classification. To bridge this gap, taxonomically annotated bulk-level training images are needed.

Capturing images of unsorted bulk samples is a straightforward procedure, yielding a single image depicting all specimens from the sample, hereafter referred to as a *bulk image*. Classifying insects from such bulk images presents two key challenges. First, given insects’ small size and the high specimen density in bulk samples, individual specimens appear as tiny objects within the image, with restricted morphological detail. Second, insects’ high taxonomic diversity requires large training datasets to achieve adequate coverage of different taxa by the classifier. Together, these pose a significant challenge for the taxonomic annotation of images. As a consequence, previous studies attempting insect classification from bulk images have worked with a substantially reduced number of classes^11,12^, resulting in more coarse information than what is often needed for community ecology.

DNA barcode data can also be generated from bulk samples, a process called DNA metabarcoding^13^. The resulting DNA barcodes can then be classified into taxa by comparison with extensive reference databases, such as the Barcode of Life Data Systems (BOLD)^14^. Assuming that the target group is well-represented in the reference data, DNA metabarcoding produces detailed information about the taxonomic composition of the sample. It also yields data on the relative abundances of different sequence variants. However, one of the main drawbacks of DNA metabarcoding is that it is challenging to infer absolute abundances across samples and taxa^15^. To address this challenge, we propose to integrate DNA and image data, leveraging the strength of both modalities. Combining detailed taxonomic classifications from DNA metabarcoding with bulk images of the same samples, where counting of taxa is relatively straightforward, could enable absolute, taxon-specific abundance estimates. Using both images and barcodes has been shown to enhance classifier performance compared to using either data source in isolation^16,17^. Further, when using individual-level data of both modalities, the genus of unknown species was predicted with more than 80% success^16^. This could allow researchers to explore the taxonomic composition at higher ranks even in samples which contain a large number of unknown species, thus enabling studies of particularly diverse ecosystems and organism groups. An additional benefit of combining molecular and image data is the potential to extract trait data, such as the body size distribution across individuals in a sample^11^.

Here, we present the Mixed Arthropod Sample Segmentation and Identification (MassID45) dataset, centred on 45 bulk arthropod samples (primarily insects) collected using Malaise traps deployed in Sweden and Finland in 2021. Harnessing both imaging and molecular information, each sample features DNA metabarcoding data and one or more unsorted bulk images. We further provide sample-level biomass measurements, which may support trait-based analyses. To facilitate the training of machine learning-based classifiers, we additionally supply individual-level images and DNA barcode sequences obtained after sorting each sample into individual specimens (35510 in total). Leveraging AI-assisted annotation and sample-specific DNA-based classifications, we provide a detailed segmentation mask and taxonomic annotation for each arthropod in the bulk images. Using the bulk-level data, we benchmark instance segmentation on tiny objects, drawing on related methods for object detection. Our dataset and experiments provide a valuable perspective on the domain-specific challenges of capturing tiny, densely arranged, and sometimes overlapping objects — an area less emphasized in standard object detection datasets. Recent advances in tiny object detection^18,19^, open-world detection^20^, multi-modal learning^16,17^, and AI-assisted annotation^21^ collectively allow researchers to better detect, classify, and analyze numerous minuscule specimens in a single frame. This dataset is poised to support a wide range of ecological applications, such as training automated classifiers for bulk samples, accurately counting specimens in large collections, and enabling large-scale morphological analyses. From a machine learning standpoint, it also opens up avenues in developing instance segmentation methods for tiny objects, exploiting weakly labelled information for classification, and open-world detection using fine-grained classes. By bridging these fields, our resource provides a valuable foundation for further research in both ecology and machine learning.

## Methods

### Spatiotemporal sampling information

**Fig. 1:**
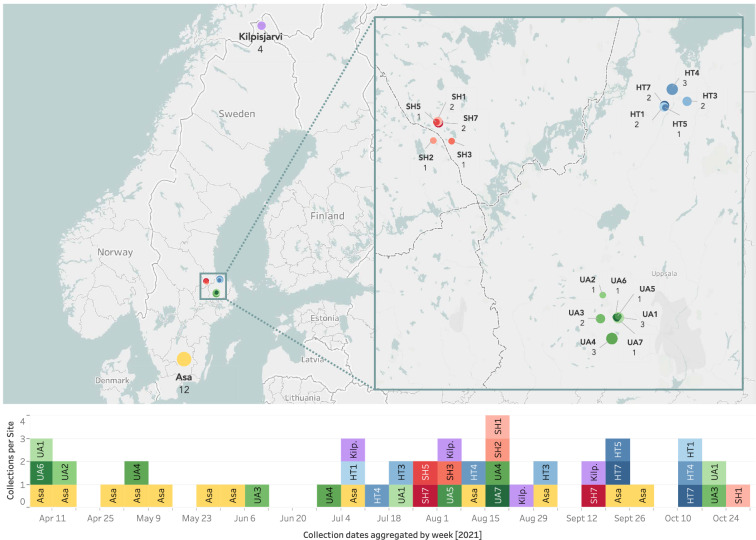
Geographical distribution and collection dates of samples. Top: Locations of the 19 sampling sites across Sweden and northern Finland. SH, HT, and UA are part of a hierarchical sampling design, each including 5–7 trap locations. The size of each circle is proportional to the number of samples collected at that site, which is also indicated by an integer below the trap name. Bottom: Temporal distribution of the MassID45 samples. Collection dates have been aggregated by week so that samples collected during the same week are displayed in the same column, regardless of what day of the week they were collected. Hierarchically organized sites (SH, HT, and UA) are coloured with shades of the same main colours to emphasize their geographical proximity.

We sampled arthropod communities at 19 sites in Sweden and northern Finland using Townes-style Malaise traps^22^. We deployed the traps continuously during 2021 and emptied them once per week. The MassID45 dataset we present here constitutes a subset of 45 of these samples, collected between March 31 and October 25, 2021 (Fig. 1). Each sample is uniquely named with a six-character alphanumeric code and has associated geographic and temporal information, including the latitude and longitude of the sampling site, as well as placement and collection dates. 17 of the 19 sampling sites were part of a hierarchical sampling design^23^ and were therefore clustered relatively closely together. In ecological analyses, this nested design allows us to compare samples which are close to each other (some 10^2^ m) to sites which are farther away (10^6^ m) through a range of scales (Fig. 1). After collection, samples were shipped to the Centre for Biodiversity Genomics, Guelph, Canada, where they were preserved in fresh 96% ethanol and stored at −20^°^C until analysis.

### DNA barcoding and imaging workflows

#### Bulk sample analysis

**Fig. 2:**
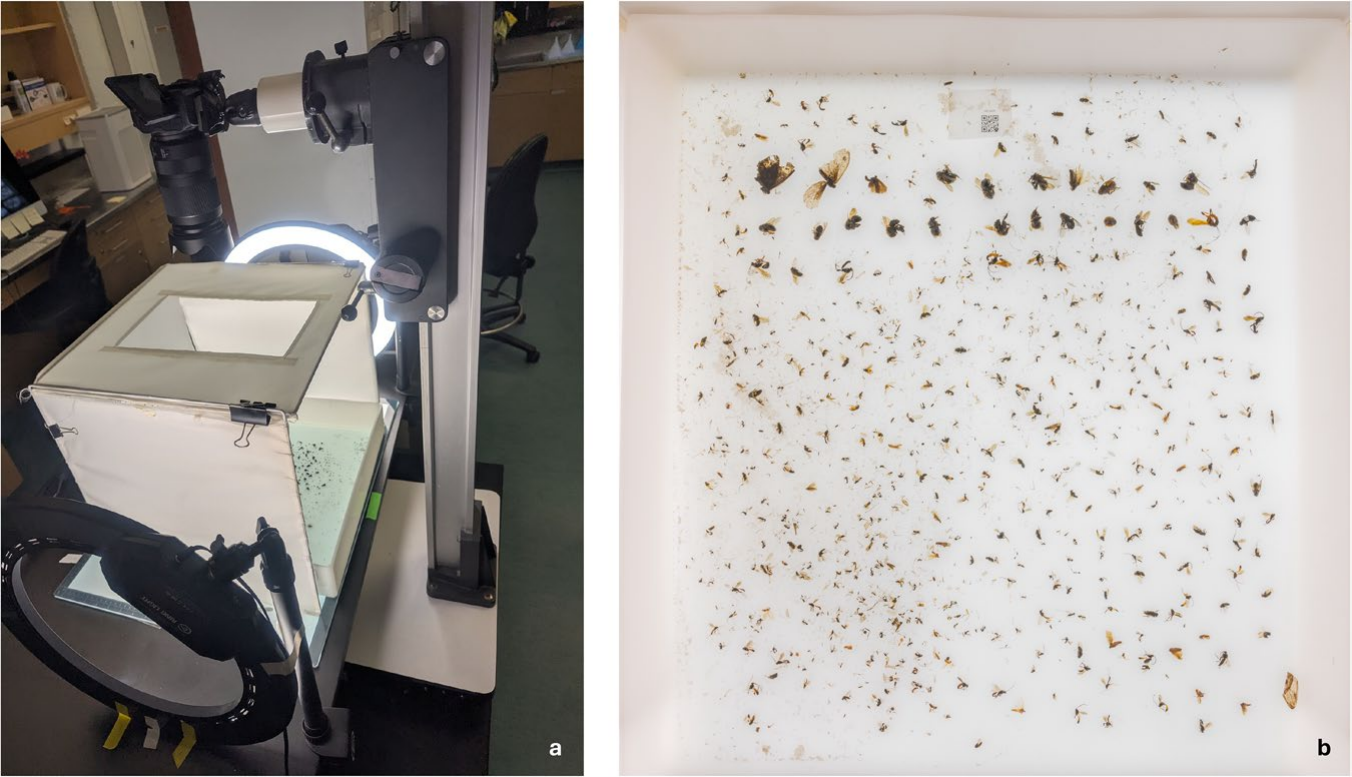
**(a**) Imaging setup used to capture bulk images of the MassID45 dataset, including the positioning of the camera, light cube and ring light sources. (**b**) A representative image captured using the described imaging setup, with the sides trimmed.

We first analyzed samples through a bulk workflow^24^, where all specimens collected in a sample were analyzed simultaneously without prior sorting. We weighed the arthropods from the bulk sample after filtering out the ethanol to obtain the wet biomass. We then performed non-destructive lysis for DNA extraction and collected three technical replicates from each sample. After extraction, we amplified a short (418 bp) fragment within the standard barcoding region of COI, which we then sequenced on an Illumina NovaSeq 6000. After DNA extraction, we transferred each sample to a translucent sorting tray (44 × 39 cm) with a shallow layer of ethanol and carefully spread out the specimens to minimize overlap. We placed the tray on an LED panel inside a modified light cube, where the front panel was removed and a hole was added in the ceiling to fit a camera (Fig. 2a). To further improve light conditions, we used two ring lights placed on opposing sides of the light cube. We captured a bulk image from above with a Canon EOS R5 camera and an RF 24–240 mm F4-6.3 IS USM zoom lens mounted on a large copy stand. We used the following camera settings: focal length of 27 mm, aperture f/20, shutter speed 1/6 seconds, and ISO 100. Each photo included a QR code unique to the sample (Fig. 2b). For four samples weighing more than approximately 10 g, we divided the sample into two sorting trays, resulting in two bulk images for each of these samples, for a total of 49 images.

We manually edited the full-resolution RAW images (45 megapixels; 8192 × 5464) in Adobe Lightroom Classic to improve contrast and ensure visibility of both light and dark insect body parts, using the following settings: we increased exposure by 1.3 stops, set whites and highlights to -100, and shadows to +50. To restore image contrast and colour we also adjusted clarity and saturation to 20 and increased the white balance from 4200K to 5050K. To reduce noise and purple fringing, we applied luminance noise reduction and defringe values of 20. Finally, we increased sharpening to 60 and saved the images in JPEG format.

#### Individual specimen analysis

After bulk imaging was completed, we placed each specimen from the bulk samples in a separate well in a 96-well round-bottom microplate for individual analysis. Specimens smaller than 5 mm were placed directly in the well and imaged using a Keyence VHX-7000 Digital Microscope system^10^. For larger specimens (approximately  > 5 mm), we removed a single leg for DNA extraction and pinned the main body of the arthropod for imaging using an automatic Imaging Rig^25^. We amplified and sequenced full 658-bp DNA barcodes for each specimen using single-molecule real-time (SMRT) sequencing^26^ on a PacBio Sequel platform. The success rate of amplification and sequencing of DNA barcodes from the individual specimens was 97.5%, though only 89.6% passed quality and contamination checks. In combination with factors affecting metabarcoding success, such as primer bias and amplification of non-target DNA (e.g., gut contents), we therefore expect some discrepancies between the individual and bulk-level DNA barcoding data. We uploaded images and DNA barcodes to BOLD and assigned taxonomic classifications based on both image and molecular information using the BOLD ID engine. We retained all specimens for future morphological reference in the natural history collection of the Centre for Biodiversity Genomics (BIOUG).

*Sample-specific taxonomies*. Using individual-level DNA barcodes, we constructed sample-specific taxonomies to guide the annotations of the corresponding bulk images. To do this, we used a base taxonomy covering all arthropods in Sweden, which we then subset for each sample to include only taxa observed in that sample. We created the base taxonomy containing the ranks kingdom, phylum, subphylum, class, order, suborder, infraorder, superfamily, family, subfamily, genus, and species, by starting with the taxonomy provided by BOLD^14^. We supplemented the BOLD taxonomy with the ranks suborder, infraorder and superfamily from Dyntaxa, the Swedish taxonomy database (downloaded from https://artfakta.se/), which covers all arthropods recorded in Sweden. We subset the Dyntaxa taxonomy to Hexapoda and Arachnida and combined it with the BOLD taxonomy by matching genus names within phyla and classes. Any taxonomic discordances between the taxonomies were resolved by giving the BOLD taxonomy precedence as follows. We used family names as they occurred in the BOLD taxonomy. For cases where Dyntaxa used a different family name, we verified that the suborder, infraorder, and superfamily from Dyntaxa still applied to the BOLD family name by comparison with the NCBI taxonomy database^27^. If NCBI listed another suborder, infraorder, or superfamily for the BOLD family name, we changed the discordant rank in our taxonomy to match NCBI. However, we did not add any information from NCBI to ranks that were empty in our taxonomy. If there was no taxonomic information for the BOLD family, we kept the information from Dyntaxa for suborder, infraorder, and superfamily. Diplura, Collembola, and Protura occurred as classes in BOLD but as orders in Dyntaxa. We kept them as classes in our taxonomy and removed all sub- and infraorders, as the same taxa appeared as orders in BOLD. The orders Phthiraptera and Psocoptera in the Dyntaxa taxonomy were combined into the order Psocodea in BOLD. We therefore used the latter in our taxonomy. We also added “microlepidoptera” as an informal taxonomic group between the ranks of infraorder and superfamily. This group included 14 superfamilies within the order Lepidoptera (Adeloidea, Choreutoidea, Gelechioidea, Gracillarioidea, Micropterigoidea, Nepticuloidea, Pterophoroidea, Pyraloidea, Schreckensteinioidea, Tineoidea, Tischerioidea, Tortricoidea, Urodoidea, and Yponomeutoidea). While microlepidoptera is not a true taxonomic group, it is a useful classification when working with insect images with low resolution. Finally, we generated sample-specific taxonomies by using the taxonomic classifications obtained from DNA barcoding of individual specimens in each of the 45 samples to subset the base taxonomy.

### Bulk image annotation

We annotated the bulk images in two steps: first, creating segmentation masks around each visible specimen, and second, assigning taxonomic labels to each arthropod mask. This workflow confined the need for taxonomic expertise to the second step. Our approach combined three key strategies to facilitate annotation: watershed segmentation for generating initial masks^11^, the Toronto Annotation Suite (TORAS)^28^ for AI-assisted mask refinement, and the DNA barcoding-derived sample-specific taxonomies to restrict the set of suggested taxonomic labels to taxa that were confirmed to be present in each sample. The complete annotation workflow is described in detail below.

#### Annotation step 1: Create segmentation masks

To facilitate rapid annotation of a large number of arthropods, we used a watershed algorithm to generate initial segmentation masks^11^. Watershed segmentation treats the image as a topographical surface, where pixel intensities represent heights, and finds boundaries between regions by simulating water filling up from local minima. Contiguous areas with pixel values below a threshold (200 in 8-bit grayscale) were grouped into a segmentation mask. Although computationally efficient, this simple algorithm often merged close groupings of arthropods into single masks and excluded light-coloured or translucent body parts, such as wings, and slender structures, like legs and antennae, from the masks. Therefore, we improved the masks by manually editing them using TORAS, a web-based annotation tool harnessing human-in-the-loop AI models to speed up and improve annotations for computer vision.

To permit fast annotation of tiny objects, we implemented two custom features in TORAS. First, we modified the default zoom behaviour when creating a new segmentation mask: instead of zooming out to show the full image, we maintained the current zoom level to prevent the annotator from losing track of individual insects. Second, we integrated a scale bar to help annotators better gauge object sizes within the images. Further, since the bulk samples contained between 36 and 3228 arthropods each, we used a custom script to split images into subimages for faster mask rendering in TORAS. We first split images into 4 × 4 equally-sized subimages. To avoid splitting arthropods across subimage boundaries, we used the initial watershed masks to locate them. We calculated each mask’s centroid and assigned it to the corresponding subimage. We then adjusted subimage sizes to include the full range of each initial segmentation mask, with a 100-pixel buffer to allow for manual mask adjustments. This method of splitting resulted in some overlap, causing some arthropods to appear in multiple subimages. We visually tagged these arthropods in all but one of the subimages they appeared in to prevent redundant manually created masks.

We uploaded the initial segmentation masks to TORAS and automatically improved them with the built-in segmentation refinement tool, which adjusts each mask to include only pixels belonging to the object. Compared to the raw watershed masks, the automatically refined masks provided a more precise fit around the arthropods. Three annotators with basic knowledge of insect morphology then manually edited the refined masks to ensure that each arthropod had a separate segmentation mask, capturing all its pixels without including any background. The annotators could utilize the full range of tools available in TORAS for editing the masks. For example, Paint and Erase were commonly used to manually edit the masks, while the Box Tool, which estimates a segmentation mask based on a bounding box provided by the annotator, was used to generate masks for arthropods missed by the watershed algorithm. Finally, the annotators assigned each segmentation mask to one of four coarse classes: arthropod (b for “bug”), debris (d), edge (e, including tray edges and QR codes) or unidentifiable (u). For detailed annotator instructions, see Supplementary Information S1.

#### Annotation step 2: Assign taxonomic labels

In the second step of annotations, one “expert annotator” with experience in arthropod identification used TORAS to assign taxonomic labels to each segmentation mask previously classified as containing an arthropod (class b). To minimize misspellings or taxonomic disagreements between ranks, the expert annotator selected labels from the sample-specific taxonomy constructed from the individual-level DNA-based taxonomic classifications (see Individual specimen analysis). However, since there was some, albeit small, proportion of specimens for which the individual DNA barcoding failed, the expert annotator was able to override the default set of choices by creating and assigning labels that did not occur in the sample-specific taxonomy. The expert annotator was asked to assign the lowest taxonomic group possible for each segmentation mask containing an arthropod.

To allow the expert annotator to convey as detailed taxonomic information as possible, we distinguished between labels of high and low confidence using two different methods. First, by assigning multiple taxonomic labels at different ranks to a single segmentation mask: the label which belonged to the highest taxonomic rank was considered high confidence, while all lower-ranking labels were treated as low confidence. That is, if the expert annotator assigned order:Lepidoptera and family:Tortricidae, Lepidoptera was considered high confidence and Tortricidae low confidence. Second, by assigning multiple labels at the same rank: their most recent common ancestor was considered a high-confidence label, while all other labels were treated as low-confidence. For example, if the expert annotator assigned both family:Tortricidae and family:Geometridae, these family labels would be considered low confidence, while their most recent common ancestor, order:Lepidoptera, would be interpreted as high confidence, regardless of whether the expert annotator explicitly assigned it. These methods allowed the expert annotator to assign uncertainty without requiring a separate step for assessing label confidence. They also avoided the difficulty of the expert quantifying their confidence.

In addition to assigning taxonomic labels, we asked the expert annotator to perform a quality check of the segmentation mask from the first annotation step. This was done with a custom feature in TORAS, which allowed annotators to view the segmentation masks in the second annotation step. During the quality check, the expert annotator primarily adjusted the mask if visual characteristics important for the classification of the arthropod, such as wings or antennae, were not included in the mask, or if multiple arthropods were grouped in one mask. The expert annotator could also change the initial assignment to one of the four coarse classes, if, for example, debris or an unidentifiable object had mistakenly been labelled as an arthropod. For detailed annotation instructions, see Supplementary Information S1.4.

#### Annotation completeness and reliability

We were able to annotate the majority of specimens in the bulk images at rank suborder or above with high confidence (Table 1). Including low-confidence annotations increased the number of specimens annotated at lower ranks, with almost half of the specimens annotated at the superfamily level, and more than a third at the family level (Table 1). At lower ranks, such as genus or species, taxonomic classifications from bulk images alone become practically impossible for most taxa. Morphological characteristics may be obscured by the random orientation of insects in the images, where, for example, wings can be hidden under the main body, or by the low resolution at which individual specimens are depicted within the bulk images. Additionally, many diagnostic features are only available through microscopy or dissection of structures such as the genitalia, making examination of the physical specimen effectively necessary. The strength of this dataset thus lies primarily in its utility for training models that operate at the order or family level, where label coverage and confidence are highest. Even without species-level resolution, these ranks are often sufficient to reveal broad patterns in biodiversity and community composition^29,30^. In the bulk images, 45 specimens were annotated with the short label b without any additional taxonomic label, resulting in 17892 of 17937 bug segmentation masks being taxonomically labelled.

**Table 1:** Number of unique taxa and specimens annotated at each taxonomic rank. Values are shown separately for annotations of high-confidence (HC) and annotations of low-confidence and above (LC).

Rank	# Taxa		# Labelled		Labelled (%)	
	HC	LC	HC	LC	HC	LC
Phylum	1	1	17892	17892	100.0%	100.0%
Class	4	4	17570	17834	98.2%	99.7%
Order	23	25	15042	16463	84.1%	92.0%
Suborder	8	8	11218	12745	62.7%	71.2%
Infraorder	5	6	7792	10778	43.5%	60.2%
Superfamily	34	47	6261	8730	35.0%	48.8%
Family	92	129	4546	6358	25.4%	35.5%
Subfamily	27	36	993	1174	5.5%	6.6%
Genus	35	55	584	694	3.3%	3.9%
Species	17	23	63	74	0.4%	0.4%

Within this work, we did not perform any independent validation of annotations beyond the sanity check of segmentation masks made during the second annotation step, primarily due to limited annotator availability during dataset development. This makes it difficult to directly evaluate the consistency and uncertainty associated with both the segmentation masks and the taxonomic annotations. However, the combination of bulk-level and individual-specimen data in our dataset provides an internal basis for comparing annotated labels against expected patterns, which can give an indication of annotation reliability.

**Fig. 3:**
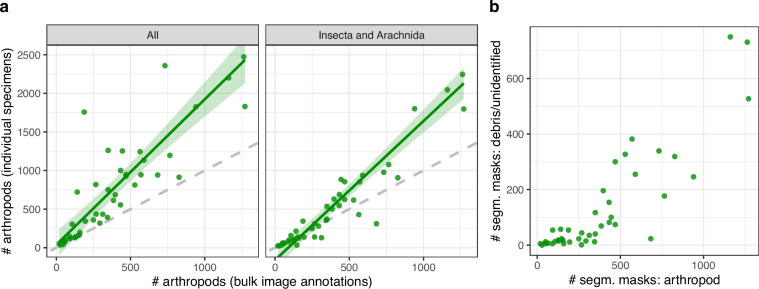
(**a**) For each sample, a comparison was made between the number of arthropods annotated in the bulk images and the number of individual specimens isolated from the corresponding samples, here shown for all taxa (left) and restricted to Insecta and Arachnida (right). The green line represents a linear regression fit with a 95% confidence interval between the two quantities, and the dashed grey line indicates a 1:1 relationship. (**b**) For each sample, a comparison was made between the number of segmentation masks tagged as arthropods and the number tagged as debris or unidentifiable across all bulk images.

To evaluate how accurately the bulk image annotations reflect the true number of arthropods in the samples, we compared the number of segmentation masks annotated as arthropods with the actual number of specimens isolated from each sample (Fig. 3a). We found that in samples containing more than approximately 250 arthropods, the number of arthropods based on the bulk image annotations was substantially lower than the true count. Some of these discrepancies occurred in samples with a high abundance of springtails (Collembola), which are often small, pale, and difficult to separate from debris in the bulk samples. Restricting the comparison to individual specimens classified as Insecta or Arachnida (both of which are typically larger and darker than springtails) reduced the difference between annotated counts and true specimen counts. Overall, the absolute discrepancy in counts tended to increase with larger samples, suggesting two possibilities. First, it is inherently challenging for human annotators to detect all insects in images where the total number of individuals is very high. These samples also often contained substantial debris, which can obscure smaller insects (Fig. 3b). Additionally, because each insect occupies only a small proportion of the image, especially tiny insects may appear visually indistinct or blurry, making them difficult to annotate correctly. This was particularly prominent when insect sizes varied greatly within an image, as the limited focal plane prevented all insects from being equally in focus. Second, annotator fatigue may set in for these large samples, leading to fewer corrections for arthropods missing a segmentation mask once the total count is already high. However, this explanation remains speculative as we did not record the annotation session metadata necessary to test the effect of fatigue on annotation rates directly. Recording such metadata in future dataset efforts would enable more rigorous quality assessment in this respect.

**Fig. 4:**
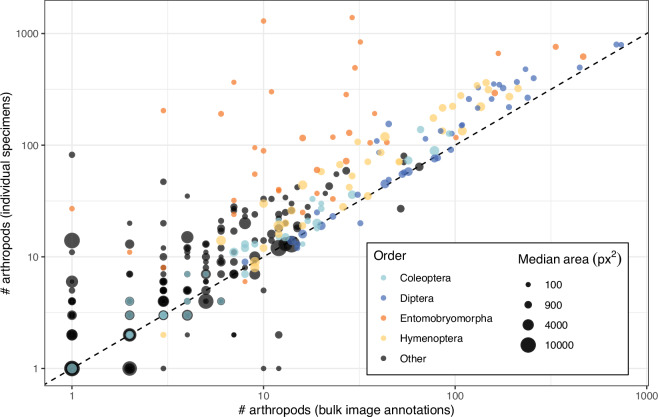
For each sample and order, we compared the number of arthropods annotated in the bulk images and the number of individual specimens isolated from the corresponding samples. The dashed line shows a 1:1 relationship between the two counts. The areas of the circles represent the median segmentation mask area for each sample and order. The four most abundant orders in our datasets are shown in colours, while all other orders are grouped together and shown in black.

Using high-confidence taxonomic annotations at the order rank, we compared the number of bulk image annotations with the number of specimens isolated from each sample (Fig. 4). We found that the bulk image annotations generally recovered fewer specimens per order than sorted individuals, but that the proportion of specimens missing from the annotations was largely consistent across sample sizes, suggesting that the relative proportion of taxa is reliably captured in our annotations. In line with the total count comparison, Collembola – represented here by the largest order, Entomobryomorpha – were the exception, with many specimens missing from the bulk image annotations. This pattern may again reflect that many of the smallest specimens were missed during annotation due to their poor visibility. However, the size distribution of our bulk image annotations overall agrees with the expected distribution from Malaise trap catches. Here, the “tiny” category corresponds to specimens with an annotated area of <1 mm^2^. Translating the annotation area to conventional body size is not straightforward, as the measured area may be inflated by structures such as wings, and body length cannot be directly inferred from area. Nevertheless, 60% of insects in our dataset had an annotated area below 3 mm^2^, which is similar to size distributions reported from other Malaise trap collections^31^.

### Machine learning dataset

**Fig. 5:**
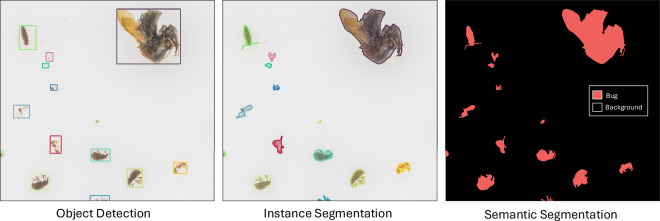
Differences between annotations for object detection (left), instance segmentation (middle), and semantic segmentation (right).

MassID45 serves as a benchmark dataset for instance segmentation of tiny, densely packed objects. Instance segmentation differs from other tasks like object detection, which uses bounding boxes, and semantic segmentation, which labels pixels but does not distinguish between individual objects. In contrast, instance segmentation assigns a unique pixel-wise mask to each individual object (Fig. 5). Using the fully annotated set of 49 bulk images, we limited the task to instance segmentation of arthropod specimens (class b), i.e., excluding objects tagged as debris or unknown. This allowed us to approach this problem as a single-class, small-instance segmentation task.

**Fig. 6:**
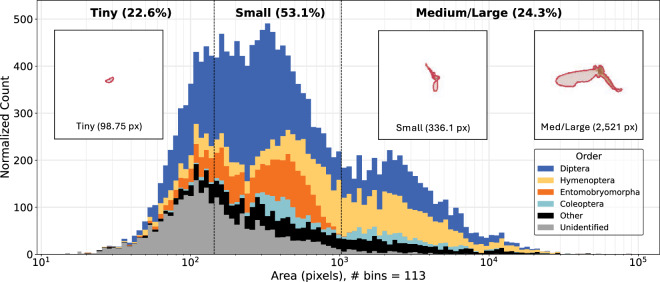
Distribution of insect mask areas for “tiny” (<144 pixels), “small” (≥144 but <1024 pixels), and (“medium/large” (≥1024 pixels) insects. Counts are adjusted such that the area of a bar is proportional to the count in that bin. Colours within the stacked bars represent the four most abundant orders, all other named orders grouped as “Other”, and orders without order-level annotation as “Unidentified”. The three overlaid images show the median masks for tiny, small, and medium/large insects, all at the same magnification.

Annotations are exported from TORAS in the same format as the Microsoft Common Objects in Context (MS-COCO) dataset^32^, a benchmark dataset for pretraining object detection and instance segmentation models. We based the evaluation scheme for the instance segmentation models on MS-COCO conventions, including metrics that rely on the areas of the instance masks to be detected. However, the MS-COCO evaluation scheme was designed for objects that are significantly larger than those in the MassID45 dataset, with 76.5% of the arthropod masks being categorized as “small”. Therefore, to increase the granularity in our performance evaluations, we instead used the area thresholds from iSAID^33^, a dataset of remote sensing images intended for small object detection and instance segmentation. Thus, we defined object sizes as follows: “tiny” for areas  < 144 pixels; “small” for areas ≥144 but  < 1024 pixels; and “medium/large” for areas ≥1024 pixels. In total, the 49 fully annotated bulk images contain segmentation masks for 17937 arthropods. Mask areas range between 15.1 and 83182.4 pixels, with a mean and median of 1152.2 and 343.4 pixels, respectively (Fig. 6).

### Preprocessing bulk images

Before training deep neural networks to perform instance segmentation, we preprocessed the image and segmentation mask data. We first merged the annotations from the subimages back together to match the original bulk images (Bulk image annotation). This allowed us to break up the images into tiles as needed for model training, detailed below. We used the Shapely library^34^ in Python to merge segmentation masks with multiple polygons and to correct invalid (i.e., self-intersecting) polygon masks. The insect masks were processed as concave hulls, filling in holes (e.g., areas between legs) in the edited segmentation masks to create single polygons. For 63 of the 17937 insect masks (0.351%), these preprocessing steps resulted in several unconnected polygons that could not be merged via a unary union. In such cases, we took the polygon with the largest area as the final mask. This ensured that deep learning models only needed to predict one polygon per annotation, simplifying the segmentation task. After cleaning the segmentation masks, we manually cropped the bulk images to only contain the areas in which insects were present. This resulted in finalized cropped bulk images of different dimensions.

## Data Records

The MassID45 dataset is organized into two resolution levels (Table 2): bulk samples containing bulk images, metabarcoding data, and taxonomic image annotations and individual specimens containing individual images and DNA barcoding data. Sample metadata, bulk sample images, bulk image annotations, and models described here are all available from Zenodo^35^. Sample metadata is provided in a CSV file with one row per sample, uniquely identified by a six-character alphanumeric code. The same sample code is used as the file name of the corresponding bulk image, followed by the suffix _{image}, where image is 1 or 2, in cases where there is more than one image per sample. We provide each image raw in CR3 format and edited in JPEG format. Bulk image annotations are available from steps 1 and 2 in both COCO and TORAS formats. The trained models are provided as PyTorch checkpoints and can be used for model inference with the code provided at https://github.com/uoguelph-mlrg/MassID45.

**Table 2:** Overview of data types included in the MassID45 dataset.

Resolution	Data type	Quantity	Description
Bulk samples *N* = 45	Bulk images	49 images (of 45 samples)	Images depicting unsorted insect samples, with 1-2 images per sample (41 samples 1:1, 4 samples 1:2).
	Metabarcoding data	45 samples	COI sequences from metabarcoding of unsorted insect samples. Each sample has three technical replicates.
	Taxonomic image annotations	17937 annotations	Segmentation masks and expert taxonomic assignments for individual arthropods in the bulk images.
Individual specimens *N* = 35510	Individual images	35510 images	Images of each arthropod specimen from the 45 bulk samples.
	Barcoding data	35510 sequences	COI sequences from DNA barcoding of individual insect specimens.

Raw sequencing reads for bulk samples are available from ENA^36^ under project accession number PRJEB86111. The sequences for each sequencing replicate are represented by two gzipped FASTQ files, containing the R1 and R2 paired-end reads. Thus, for each physical sample there are a total of six files, with names of the form {sample}_Rep{i}.R{read}.fastq.gz, where {sample} is the six-character alphanumeric code uniquely identifying the sample, {i} is an integer between 1 and 3 indicating the replicate number, and {read} is 1 or 2. Accession numbers for individual samples and read files, along with a script to download all relevant files, are provided in MassID45_ENA_accnos.tsv, and download_MassID45_ENA.sh, respectively, both available at the above GitHub repository. Individual arthropod images and DNA barcode sequences are available as project ID DS-LPEPA22 on BOLD^37^. On BOLD, the field ID variable corresponds to the sample code used in the sample metadata and bulk image names, while the sample ID is an identifier unique to each individual specimen.

## Technical Validation

In this work, we benchmark instance segmentation performance on MassID45 using two paradigms: zero-shot learning and supervised learning. This analysis allows us to evaluate how valuable the expert annotations are for detecting small arthropods, compared to “out-of-the-box” generalist models.

Under the zero-shot paradigm, we employ models that have not seen any training examples from the MassID45 data. Zero-shot models rely exclusively on their pre-training data – which includes large, diverse computer vision datasets – to generalize to images from unseen domains^38–43^. With supervised learning, we instead train instance segmentation models^44–46^ using annotated examples from the MassID45 dataset. By comparing the performance of zero-shot and supervised approaches, we can assess whether the expert annotations are valuable enough to justify the annotation effort, or whether existing generalist models achieve adequate detection performance on the MassID45 data. We describe implementation details for our training and inference pipelines below.

### Experimental setup

#### Dividing bulk images into tiles

Due to GPU memory constraints and the high resolution of the images, we could not present entire bulk images to deep learning models during training or inference. As a solution, we split the bulk images into tiles, similar to previous work^47,48^. Compared to down-sampling the images, which can also be used to produce a resolution which fits within GPU memory, tiling preserves the pixel density of the original images. Using tiling thus avoids a loss of visual details, which is particularly important for the small insects in the MassID45 dataset. We used a sliding window to crop tiles out from the bulk images, and each tile was then treated as a separate image during model training and inference. We determined the optimal tile size for training and inference to be 512 × 512 pixels (see Supplementary Information S2.1). During tiling, some insects may get cut between tiles. To mitigate this, we used an overlap of 60% between tiles, similar to previous work on small object detection^48^, thus ensuring cut insects along the boundary of one tile were shown intact in adjacent tiles.

Tiling introduces a challenge during inference: when the same insect appears in multiple overlapping tiles, treating each tile as an independent image would lead to duplicate detections and inaccurate performance estimates. To address this, we implemented slicing-aided hyper-inference (SAHI), a method designed to merge predictions across overlapping tiles and accurately reconstruct detections in the full bulk image^49^. The SAHI algorithm has previously been used for small object detection problems in remote sensing^50–52^ and pest monitoring^53^. We used SAHI to postprocess the tiled predictions by applying non-maximum merging (NMM)^54^. NMM relies on the Intersection over Union (IoU; see Evaluation metrics below), a measure of how much two masks overlap, to identify and merge predictions which are likely duplicates. After sorting predicted masks across all tiles by their confidence scores, NMM identifies and groups detections that overlap by more than a predetermined IoU threshold (*IoU*_*NMM*_). Within each group of overlapping masks, NMM iteratively merges pairs of predictions, producing a new mask that spans their combined area and a new confidence score weighted by the original masks’ confidence scores and areas. This pairwise merging continues until one mask remains for each group of overlapping detections. Lastly, we mapped the final set of merged predictions from the tiles back onto the original bulk image, allowing us to evaluate them directly against the ground truth bulk images. When merging the predictions across tiles, we used an *IoU*_*NMM*_ of 50%, meaning that overlapping predictions were considered duplicates and iteratively merged if their intersection over union was at least 50%.

#### Data partitioning

We randomly partitioned the bulk images into training (40 images, 81.6%), validation (3 images, 6.1%), and testing (6 images, 12.2%) sets. After dividing the bulk images into 512 × 512 tiles, this resulted in 17062 training tiles, 1244 validation tiles, and 1586 testing tiles. To prevent data leakage, all tiles from a given bulk image were assigned to the same dataset split. Including insects that were duplicated and/or partially cut between tiles, the tiled training set contained 110520 insects, the tiled validation set 5867, and the tiled test set 6241. The validation and test sets contained data not seen during training and served to evaluate how well the models generalized to new, real-world data. The validation set was used to guide intermediate modelling decisions, such as selecting between models or preprocessing techniques, while the test set was used to measure the performance of the final model after all model development and experimentation were complete.

#### Data augmentations

To artificially increase the number of training samples and improve generalization, we applied data augmentations to the tiled images from the training partition, drawing on prior work focused on small object detection in remote sensing and underwater imagery^48,55^. It is important to note that our tiling process also acted as a form of data augmentation, as the arthropods could be present in multiple adjacent tiles. We employed both geometric and colour-based augmentations (Table 3), which introduced variations to the bulk images while ensuring the insects could still be identified. For example, random rotations and horizontal flips mimicked the possible orientations that arthropods can assume when placed in the sorting trays. Random adjustments to brightness, contrast, and saturation were intended to make the models more robust to small differences in lighting across bulk images, as well as natural colouration differences among arthropods (e.g., in different life stages). We applied these augmentations to the tiled bulk images, then resized each augmented tile to a fixed input size of 1024 × 1024 using bilinear interpolation before presenting them to the model during training.

**Table 3:** Geometric and colour-based data augmentations used for the training data, where *p* denotes the probability of applying each transformation.

Category	Augmentation	Parameters
Geometric	Random horizontal flip	*p* = 0.5
	Random rotation	{0^°^, 90^°^, 180^°^, 270^°^}, *p* = 0.25 each
Colour	Random brightness	Uniform in range [−15%, +15%]
	Random contrast	Uniform in range [−10%, +10%]
	Random saturation	Uniform in range [−15%, +15%]

#### Evaluation metrics

Using the predictions merged with SAHI, we calculated evaluation metrics following the MS-COCO evaluation scheme^32^, which relies on IoU, precision, and recall. For a given instance mask prediction, IoU quantifies the overlap between the predicted instance masks and ground truth annotations:

$$\,{\rm{IoU}}\,=\frac{{TP}_{p}}{{TP}_{p}+{FP}_{p}+{FN}_{p}},$$

where *TP*_*p*_ represents the number of predicted pixels that matched the ground truth (true positives), *FN*_*p*_ denotes the number of ground truth pixels missed by the prediction (false negatives), and *FP*_*p*_ represents the number of background pixels incorrectly labelled as part of the instance (false positives). When calculating the evaluation metrics, we used a confidence threshold (*conf*_*eval*_) to filter out uncertain predictions and an IoU threshold (*IoU*_*eval*_) to define how strictly the predicted masks must overlap with the ground truth annotations to be considered correct. That is, we categorized each predicted instance as a true positive (*TP*_*i*_), false positive (*FP*_*i*_), or false negative (*FN*_*i*_) based on whether its IoU with the ground truth masks exceeded *IoU*_*eval*_. Based on the instance-level categorizations, we then calculated precision and recall.

Precision was calculated as

$$\,{\rm{Precision}}\,=\frac{{TP}_{i}}{{TP}_{i}+{FP}_{i}}.$$

It quantifies how many of the insects detected by the model were actually correct. Conversely, recall was calculated as

$$\,{\rm{Recall}}\,=\frac{{TP}_{i}}{{TP}_{i}+{FN}_{i}}.$$

It reflects how many of the actual insect specimens were detected by the model. We calculated precision-recall curves by keeping *IoU*_*eval*_ fixed and varying *conf*_*eval*_. Following the MS-COCO evaluation scheme^32^, we then calculated the average precision (AP), defined as the area under the precision-recall curve, for several *IoU*_*eval*_ thresholds. Here, we report the following aggregate metrics:

- AP_50:5:95_: mean of the AP values calculated across *IoU*_*eval*_ thresholds ranging from 50% to 95% in 5% increments.
- AP_50_: AP at a fixed *IoU*_*eval*_ of 50%.
- AP_75_: AP at a fixed *IoU*_*eval*_ of 75%.

We also measured AP_50:5:95_ for the “tiny”, “small”, and “medium/large” object categories (see Machine learning dataset), denoting them as AP_*T*_, AP_*S*_, and AP_*ML*_, respectively. We report the final evaluation metrics for each supervised baseline on the test set of six bulk images.

### Benchmarking instance segmentation models

#### Implementing zero-shot detectors

Zero-shot approaches can localize objects from a new domain without any prior fine-tuning on that domain, relying exclusively on pretraining from large, diverse datasets — including multi-modal data. We benchmark their generalization capabilities by applying them to a challenging new domain: small arthropods from the MassID45 data. For consistency, we applied the same SAHI approach using 512 × 512 pixel tiles with 60% overlap (see Dividing bulk images into tiles).

We selected methods representing different forms of zero-shot detection, including unsupervised instance segmentation (CutLER)^38^, open-vocabulary or open-set models that use text prompts (Grounding DINO^39,40^, and Florence-2^41^), as well as large, state-of-the-art, multi-modal models (Gemini 2.0 Flash)^43^. To perform instance segmentation, we paired the latter three methods with Meta’s Segment Anything Model 2 (SAM 2.1)^42^, a foundation model for image segmentation. The bounding boxes from Grounding DINO, Florence-2, and Gemini 2.0 Flash were used as prompts for SAM 2.1, producing instance masks that were used in our evaluation scheme for instance segmentation. For implementation details — including the model checkpoints and text prompts used for the zero-shot models — see Supplementary Information S2.2.

#### Implementing supervised detectors

For the supervised models, we selected three general architectures originally developed for standard computer vision datasets like MS-COCO^32^, which include millions of images of everyday objects. Here, we seek to determine whether they can be adapted to small, detailed organisms like arthropods in MassID45 when guided by expert annotations. These models include a popular baseline for instance segmentation, Mask R-CNN^44,56^, and two more recent methods, Mask2Former^46^ and Mask DINO^45^. The latter two use transformer-based architectures, an approach that has driven recent advances in computer vision^57–59^, to achieve state-of-the-art results on the MS-COCO benchmark.

All supervised models were initialized with weights from a ResNet-50 backbone pretrained on the MS-COCO dataset^32^, allowing us to leverage features from a large benchmark dataset. Although MS-COCO contains no arthropods, it includes nearly 1.5 million labelled object instances, which provides models with general-purpose visual features such as edges, shapes, textures, and colour patterns. These foundational features can then be transferred across domains, like fine-grained biological imagery. This strategy, known as transfer learning^60^, offers a practical alternative to training models from scratch (i.e., random weights). Using MS-COCO pretrained checkpoints for instance segmentation ensures that all three models start with the same baseline of visual understanding, allowing us to more fairly compare how each architecture adapts to the specialized task of segmenting small arthropods in the MassID45 dataset. Training instead with randomly initialized weights would require the models to learn all visual features from a comparatively small dataset, increasing the risk of poor generalization.

Using the Detectron2 library^56^, we fine-tuned each model for 15000 iterations with a batch size of 8 (2 images per GPU with 4 GPUs), using the AdamW^61^ optimizer with a peak learning rate of 5 × 10^−5^ and weight decay of 0.05. In all training runs, we used a one-cycle cosine annealed learning rate schedule^62^ with a warm-up period of 4500 iterations. Training was performed using four NVIDIA RTX6000 GPUs. For inference, we applied the SAHI (see Dividing bulk images into tiles) approach, dividing the bulk images from the test partition into 512 × 512 pixel tiles with 60% overlap. We then used an *IoU*_*NMM*_ of 50% to map the predictions from the tiles back to the original bulk image dimensions.

#### Performance evaluation

Without fine-tuning on the MassID45 data, the zero-shot models performed significantly worse than their supervised counterparts (Table 4). Grounding DINO, the top-performing zero-shot method, only achieved a mask AP_50:5:95_ of 27.1%, which is far below the 43.5% achieved by the Mask DINO, the best model across almost all AP metrics.

**Table 4:** Instance segmentation results on the MassID45 test set for the zero-shot and supervised baselines. For each mask AP metric, the top result per paradigm is **bolded**.

Detector paradigm	Detector	AP_50:5:95_	AP_50_	AP_75_	AP_*T*_	AP_*S*_	AP_*ML*_
Zero-shot	CutLER	22.7	40.0	22.1	0.80	18.1	59.0
	Grounding DINO + SAM 2.1	**27.1**	47.6	**27.0**	1.30	**22.6**	**66.3**
	Florence-2 + SAM 2.1	16.5	28.8	16.7	3.00	12.1	41.7
	Gemini 2.0 Flash + SAM 2.1	26.2	**50.0**	23.8	**3.30**	18.3	64.2
Supervised	Mask R-CNN	42.5	**83.1**	36.6	20.0	41.6	70.4
	Mask2Former	41.4	78.7	37.4	20.5	40.0	71.1
	Mask DINO	**43.5**	80.9	**40.1**	**21.1**	**43.5**	**73.1**

**Fig. 7:**
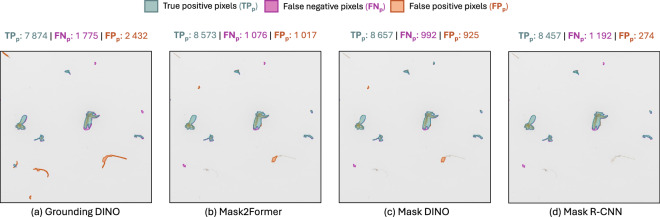
Visual instance segmentation results for one example patch from the MassID45 test set. Predicted masks are compared for (**a**) the top-performing zero-shot model, Grounding DINO; and (**b**)–(**d**) the 3 supervised baselines: Mask2Former, Mask DINO, and Mask R-CNN. Above each panel, we show the areas occupied by TPs, FPs, and FNs in pixels. Best viewed on a colour display with zoom.

It is important to note that the reported AP evaluation metrics describe performance across several IoU and confidence thresholds. When these instance segmentation models are deployed in a real-world setting, we must select a fixed operating point for the confidence threshold. For each detector, we selected distinct confidence thresholds that maximized that model’s F1-score — the harmonic mean between precision and recall — on the validation set (see Supplementary Information S2.3). We then used these confidence thresholds to filter our predictions on the test. Using these fixed confidence thresholds, we visualized predictions from each model on an exemplar patch from the test set (Fig. 7). Qualitatively, Grounding DINO could successfully localize and segment larger arthropods, but missed most small insects. It also misidentified QR codes as insects (Fig. 7a). In contrast, the supervised models produced instance masks that align well with the ground truth. However, we observed that separating debris from small insects was a difficult task, as the supervised models had a tendency to confuse small, loose debris with insects and vice-versa (Fig. 7b–d). For this exemplar patch, we also reported the number of TP, FP, and FN pixels to illustrate the differences between each model’s predictions. The zero-shot Grounding DINO model predicted significantly more FPs and FNs than the supervised models. Conversely, the three supervised models predicted similar numbers of FPs and FNs, with Mask DINO predicting the fewest FNs, and Mask R-CNN detecting the fewest FPs. Similar trends can be seen when aggregating the TP, FP, and FN pixels across the six bulk images in the MassID45 test set (see Table S2.1).

**Fig. 8:**
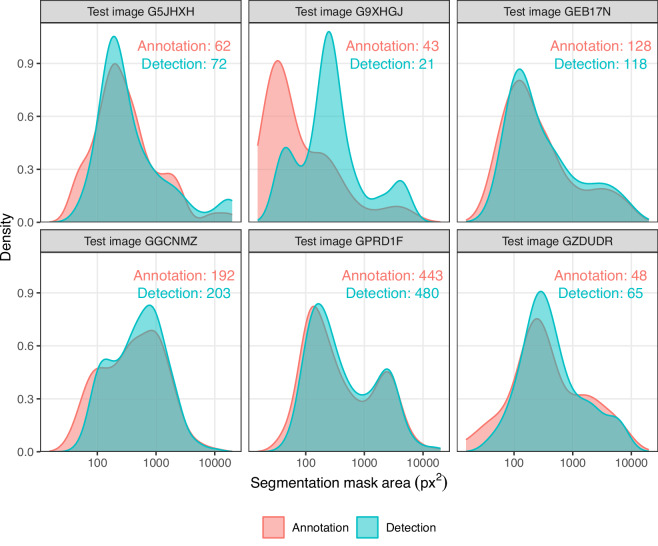
Comparison of segmentation mask size distributions between bulk image annotations (pink) and detections produced by the best-performing model (blue) on the six test images. Distributions are shown as Gaussian kernel density estimates. The text label shows the total number of arthropods from ground truth annotations and model detections for each image.

To assess the stability of our models across different bulk images in the test partition, we also calculated the AP metrics for each model by averaging across the test images (see Table 5). The number of insects in per test bulk image varied widely, with some containing only 48 insects, and others containing up to 443 insects (see Fig. 8). Thus, the image-averaged AP_50:5:95_ decreased slightly for each detector, as the AP calculations were no longer dominated by test images with many specimens. Nevertheless, the model rankings remained mostly unchanged, with Mask DINO and Mask R-CNN providing the strongest supervised performance, and Grounding DINO and Gemini 2.0 Flash achieving the strongest zero-shot performance. As expected, the supervised detectors generally demonstrated lower variance than their zero-shot counterparts, although Florence-2 achieves the lowest overall variance.

**Table 5:** To address potential variance across the six test bulk images, we calculated AP metrics separately for each image and then averaged across images (*Mean*  ± *Standard Deviation*).

Detector paradigm	Detector	AP_50:5:95_	AP_50_	AP_75_	AP_*T*_	AP_*S*_	AP_*ML*_
Zero-shot	CutLER	20.8 ± 6.90	36.7 ± 13.4	19.8 ± 6.90	2.10 ± 2.90	20.8 ± 8.90	63.0 ± 13.9
	Grounding DINO + SAM 2.1	26.3 ± 7.60	48.2 ± 16.9	25.1 ± 8.50	3.00 ± 3.70	27.3 ± 8.90	71.4 ± 10.2
	Florence-2 + SAM 2.1	15.7 ± 3.20	29.8 ± 7.60	15.0 ± 4.30	5.40 ± 4.60	16.2 ± 5.90	46.7 ± 13.3
	Gemini 2.0 Flash + SAM 2.1	26.3 ± 4.60	53.7 ± 7.50	22.0 ± 5.20	7.40 ± 4.70	23.7 ± 7.60	68.0 ± 11.6
Supervised	Mask R-CNN	41.4 ± 4.40	82.8 ± 2.90	35.5 ± 7.00	21.6 ± 9.00	43.2 ± 5.10	74.1 ± 8.10
	Mask2Former	40.1 ± 5.10	78.7 ± 5.30	34.8 ± 6.80	20.5 ± 7.80	42.3 ± 6.50	73.2 ± 10.7
	Mask DINO	41.2 ± 6.30	78.9 ± 5.60	37.5 ± 8.80	19.8 ± 8.60	43.5 ± 4.80	74.9 ± 8.60

For practical applications, individual IoU thresholds such as AP_50_ and AP_75_ are often more interpretable than the combined metric AP_50:5:95_, as they specify how closely predicted masks must align with the true insect outlines. For tasks focused on estimating specimen counts, detecting the presence of insects is usually sufficient, making AP_50_ a suitable indicator of performance. In contrast, applications such as biomass estimation require more precise delineation of insect shapes, making AP_75_ a more relevant measure of model accuracy. In our result, AP_75_ was substantially lower than AP_50_, suggesting that most masks had an IoU of less than 50%. A low IoU can result from partial detections, for example, if predicted masks are smaller or larger than ground truth annotations. From predictions on our test set, we found that Mask DINO predicts the total number of arthropods and their size distribution reasonably well, suggesting that it can accurately recover ecologically relevant data despite imperfect overlap with ground truth annotations (Fig. 8). Specimens in the “tiny” category are somewhat underrepresented in the detections, whereas larger masks are overrepresented, suggesting that MaskDINO occasionally predicts segmentation masks that are larger than the true size of small insects.

The relatively poor performance of the zero-shot baselines suggests fine-tuning is still needed for specialized tasks like detecting arthropods from the MassID45 dataset. More importantly, this finding underscores the importance of expert annotations for bulk image analyses. The complexities of the detection task are caused by the small size of the arthropods, as well as their high similarity to surrounding debris. While not explored in this work, fine-tuning these zero-shot methods on the MassID45 dataset may prove beneficial. It is important to note, however, that the supervised models explored in this work are optimized for bulk images obtained using our experimental setup and may need to be further fine-tuned on bulk images taken from different experimental conditions. Thus, this analysis frames MassID45 as a challenging benchmark dataset for custom supervised models, vision foundation models, and other zero-shot detectors, as it assesses their ability to recognize tiny, ambiguous objects rather than larger common objects that are typically considered in the literature.

## Usage Notes

Our annotation workflow consisted of two separate steps, where only a subset of the annotations from step 1 (those categorized as arthropods, b) were annotated in step 2. In step 2, the main task of the annotator was to provide a taxonomic label for each specimen. However, if the second annotator disagreed with the first categorization, they could change it to one of the three other categories (d, e, or u). For the full set of annotations with both broad categories and taxonomic annotations, the output from steps 1 and 2 must therefore be merged. For the taxonomic annotations, we used multiple labels as a way to express annotator uncertainty. If a single label is required, we therefore recommend careful selection of which taxon name to use. Because all taxonomic annotations were produced by a single expert annotator, opportunities for independent validation and inter-annotator agreement assessment were limited. Users of the taxonomic labels may therefore wish to perform additional validation depending on the intended application, especially for fine-level taxonomic analyses.

While efforts were made to ensure the bulk images were fully annotated, some insects that were at the boundaries of the 4 × 4 annotator patches may have been missed. As mentioned above in Annotation completeness and reliability, insects may appear blurry in the images. This limitation to image quality can be addressed by techniques like super-resolution, which reconstructs plausible high-quality details from low-resolution images. We leave this for future work.

For the individual specimen data, the BOLD data package^37^ includes specimens from an additional sample (GP8K9U). We have excluded this sample from the dataset we present here, as it was not sorted in its entirety.

While effective on the bulk images from the MassID45 data, the fine-tuned instance segmentation models provided in this work may not generalize to bulk images taken under different imaging protocols. Such a distributional shift would necessitate transfer learning on the user’s own set of bulk images. Nevertheless, pre-trained weights from our instance segmentation models may prove beneficial for other detection tasks involving small objects. We encourage further experimentation on the MassID45 dataset, particularly with existing instance segmentation models and vision foundation models.

## Supplementary information

Supplementary Information

## Data Availability

MassID45 sample metadata, bulk sample images, bulk image annotations, and models are available from Zenodo^35^. Raw sequencing reads for bulk samples are available from ENA^36^ under project accession number PRJEB86111. Individual arthropod images and DNA barcode sequences are available as project ID DS-LPEPA22 on BOLD^37^.

## Code availability

The code for generating the machine learning dataset and replicating our experiments is available at https://github.com/uoguelph-mlrg/MassID45.
