# Supplementary material for: A multi-modal dataset for insect biodiversity with imagery and DNA at the trap and individual level
Source: Sci Data. 2026 Apr 21;13:630. doi: 10.1038/s41597-026-07251-x (PMC13100036; doi:10.1038/s41597-026-07251-x)
Supplement: Supplementary file 1 — Supplementary Information [file 41597_2026_7251_MOESM1_ESM.pdf]

Supplementary Information

Contents

S1 Annotation instructions **1**
S1.1 Introduction . . . 1
S1.2 Getting started . . . 1
S1.2.1 Some useful terminology • S1.2.2 Data description • S1.2.3 The Toronto Annotation Suite
S1.3 Create masks + base annotations (non-expert) . . . 4
S1.3.1 Correcting masks • S1.3.2 Tips and tricks
S1.4 Create labels (expert) . . . 7
S1.4.1 The task • S1.4.2 Validate masks • S1.4.3 Classification • S1.4.4 Images of individual bugs
S2 Supplementary methods **10**
S2.1 Determining upsampling factor for tiles . . . 10
S2.2 Implementation details for zero-shot methods . . . 10
S2.3 Model evaluation with tailored confidence thresholds . . . 11
References **13**

S1 Annotation instructions

S1.1 Introduction

LIFEPLAN (https://www.helsinki.fi/en/projects/lifeplan) is a six-year initiative funded by the European Research Council (ERC) to study biodiversity in a worldwide sampling program over multiple years, using different methods to gather data across a broad range of taxonomic groups. Methods include, for example, camera traps and sound recordings. Arthropod communities are sampled using Malaise traps, a commonly used insect trap where organisms are collected in bulk and preserved in ethanol. The identification of species collected in these traps is primarily done using a technique known as DNA metabarcoding, where a short gene fragment is extracted and sequenced simultaneously for all specimens in the sample. Standardized photographs of the samples spread across a white surface are also taken and provide additional information. Further description of the arthropod data is available in section *Data description*. The objective of this project within LIFEPLAN is to develop a machine learning algorithm to classify and count arthropods from images of bulk arthropod samples.

A reliable classification algorithm for images of bulk arthropod samples has the potential to accelerate and automate insect biodiversity surveys without the need for expensive analyses. Further, it is challenging to obtain abundance estimates from metabarcoding data alone. Thus, abundance estimates and counts from images can be used as a complement or verification of estimates based on molecular data.

S1.2 Getting started

S1.2.1 Some useful terminology

To annotate an image means adding labels to it. This can be done in many different ways (for example, using points, see Figure S1.1 left). We have chosen to use segmentation masks, which means that we draw a contour around each object (arthropod, or ‘bug’) we want to label (Figure S1.1 right). When correctly annotated, each pixel that is part of that bug should be inside the mask.

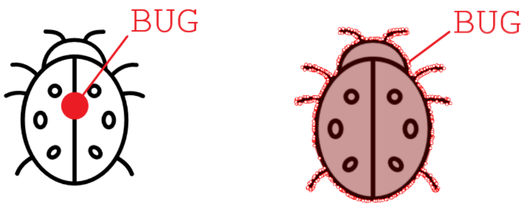

Figure S1.1. Annotation using points (left) and segmentation masks (right).

In the program we use for annotation, the different objects are called entities. Each bug should be a separate entity, which implies that each entity should contain only one segmentation mask.

Creating all the segmentation masks from scratch would take a very long time, so we use a simple method known as a watershed algorithm to generate a first draft. A watershed algorithm finds and outlines dark areas against a white background, or in our case, the different arthropods. The masks, however, are not perfect, and most need some degree of correction (Figure S1.2). The annotation task can thus be divided into two main steps: (1) create and correct segmentation masks, and (2) assign taxonomic labels to the segmentation masks. The first task requires the annotator to have basic knowledge of arthropod morphology (in this document referred to as 'non-expert'), while the second task requires detailed taxonomic knowledge (here referred to as 'expert').

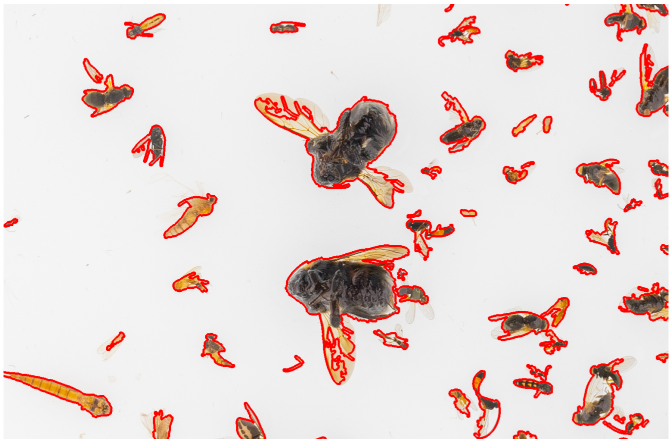

Figure S1.2. Initial segmentation masks generated by a watershed algorithm.

S1.2.2 Data description

There are four types of data relevant for this project, generated by either bulk analysis of samples (DNA metabarcoding data and bulk images) or analysis of individual specimens from the same samples (individual DNA barcoding data and individual images, here also known as 'Keyence images').

In general, in the wider Lifeplan project, Malaise trap samples are treated in "bulk", i.e. DNA is extracted from the full sample (all individuals together) at once. The resulting broth is then sequenced for a diagnostic fragment of the COI gene region, a process called metabarcoding. After metabarcoding, samples are also photographed in bulk, i.e. all individuals in one image. For this purpose, the content of the bottle is spread out on a white tray, in an effort to separate the individual bugs as well as isolate the debris. Depending on the quantity of arthropods in the sample, multiple trays may be needed, resulting in more than one bulk image per sample. As a result of this workflow, each bulk image (i.e., a picture of tens to thousands of arthropod individuals) is associated with a list of DNA-based taxonomic units ("species"). Nonetheless, since the sample is treated as one entity, individual entries within the list cannot be associated with individual arthropods in the photograph.

In this project, we are working with a subset of 45 samples where all arthropod specimens, in addition to the bulk analyses, were also individually analyzed. The individual arthropods were picked from the original bulk samples and placed in separate wells in a 96-well plate. For arthropods that were too large for wells, a leg was extracted from the specimen and placed in the well. Each specimen was then individually barcoded, i.e. the barcode region of the COI gene is sequenced. This is largely the same region as is targeted in metabarcoding, but it is possible to analyze slightly longer sequences (around 650 base pairs or positions, as compared to around 420 base pairs in metabarcoding). After molecular analyses, the arthropods were individually photographed (these images are also referred to as Keyence images). Arthropods that were too large to fit in the wells were pinned and photographed separately. Contrary to the bulk analysis, this procedure results in a set of specimens with a direct association between the DNA identification and an image.

Arthropod samples can contain around 3000 individual arthropods. To enable fast rendering of segmentation masks in TORAS, we have chosen to split images into 4×4 subimages for annotation. To ensure that no arthropods were split between two subimages, we used the initial watershed masks to indicate the location of the arthropods in the images. We calculated the centroid of each mask and assigned the mask to the subimage containing the centroid. We then adjusted the size of each subimage to include the full range of each segmentation mask assigned to that subimage, plus a buffer of 100 pixels to allow for extending the mask in any direction during manual editing (see Figure S1.3). This method of splitting images resulted in some overlap between subimages, where arthropods appeared in more than one subimage. To reduce the risk of annotators marking the same arthropod twice in two different subimages, arthropods that appeared in a different subimage than where they were originally assigned were marked by displaying the segmentation mask in the subimage.

S1.2.3 The Toronto Annotation Suite

The annotation is done in the Toronto Annotation Suite (TORAS), a web-based annotation platform. Create a free account and log in here: https://aidemos.cs.toronto.edu/toras/login. Send your username to the administrators of the project, and they will add you to the correct project on TORAS.

To get started with annotations, choose `GO TO YOUR TASKS PAGE`.

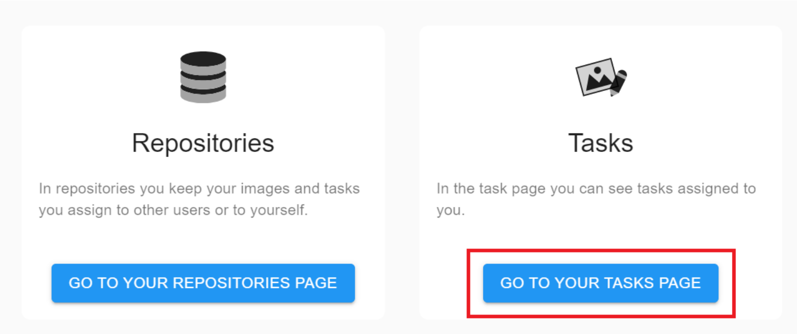

There, you can see all your assigned tasks (that is, images to annotate) and their status. Click `SELECT` to get started with a task.

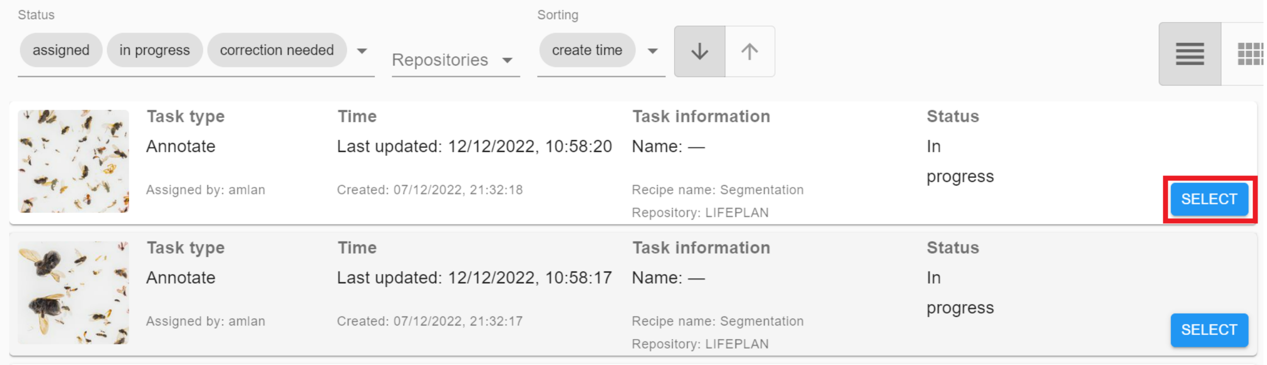

This is what the annotation page looks like:

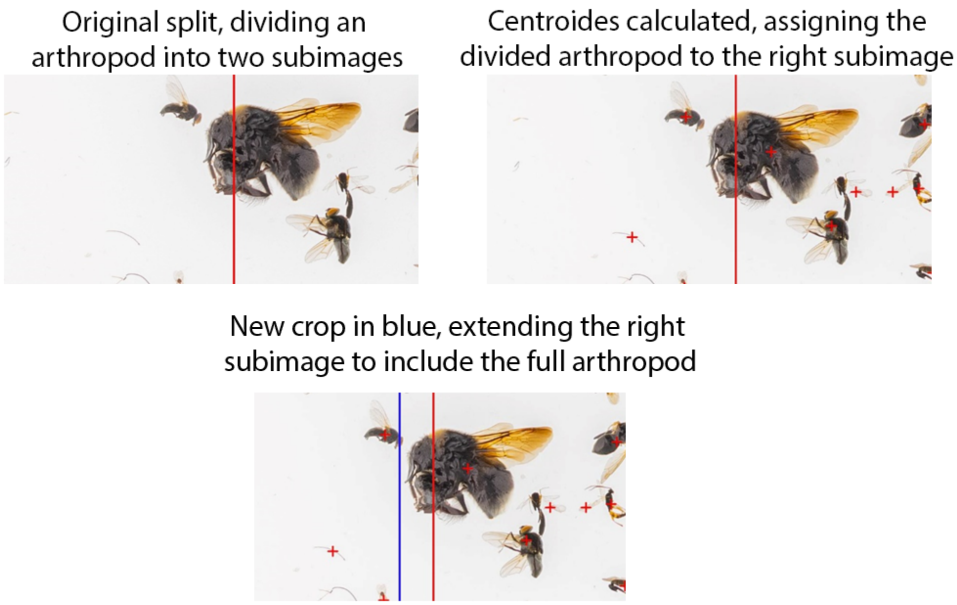

Figure S1.3. When images were split into subimages for annotation, the exact location of the split depended on the location of arthropods, to ensure that arthropods were not divided into multiple subimages.

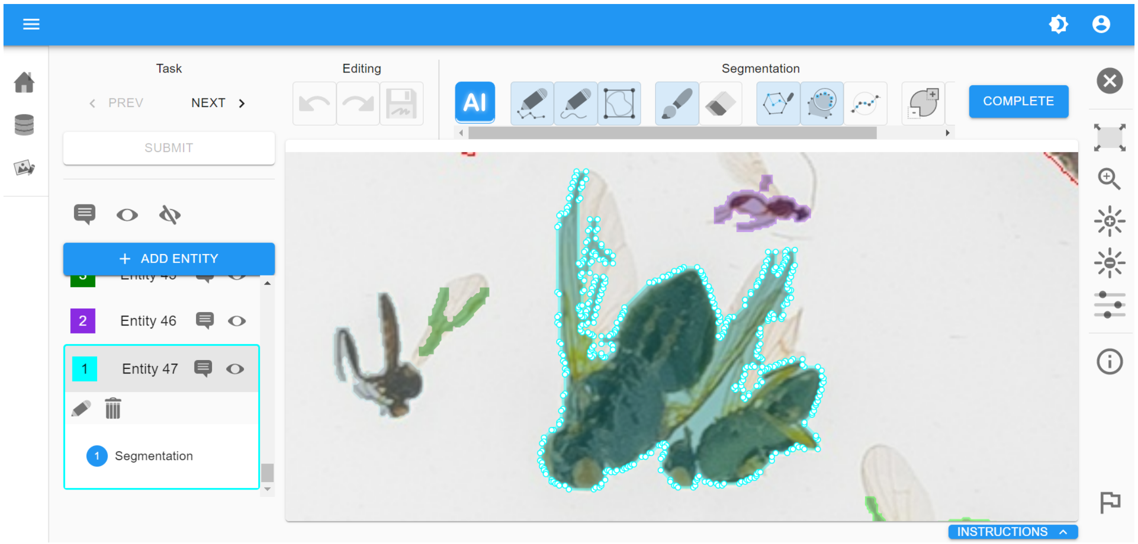

In the right panel, you have different options for zooming in/out, as well as changing brightness, mask opacity, etc. In the left panel, there is a list of the different entities in the image, each with one segmentation mask. You can zoom in on a segmentation by clicking on the entity name.

For a brief tutorial on how to use the different segmentation tools, visible in the top panel, please see this video.

S1.3 Create masks + base annotations (non-expert)

S1.3.1 Correcting masks

When images are uploaded to TORAS, they have associated masks that have been created with the watershed algorithm. The masks are automatically refined with a TORAS algorithm, but still need manual adjustments (for example, to include legs, or to remove background from the mask) and quality checks:

1. For each polygon mask:

   a. Change entity name (shortcut: `n`) to one of these short names:

Short name	Meaning	Explanation
b	bug	Any arthropod
u	unknown	Could be an arthropod, but I can't tell from the image
d	debris	Any debris, including e.g. loose legs, wings, etc.
e	edge	Mask containing tray edge, QR code, etc. i.e., not debris, not an arthropod

   b. For each mask identified as `b`, correct the mask:

      i. Include all body parts, such as legs, antennae, and wings (e.g. using the painter, shortcut: `r`). The TORAS algorithm is also good at estimating segmentation masks, so if the mask is very poor, it is sometimes faster to delete the mask and draw a new one using the bounding box tool (shortcut: `b`).

      ii. Exclude areas with only background, e.g. between legs (e.g. using the eraser, shortcut: `e`). See Figure S1.4: in the left panel, too much background is included. In the middle panel, the background between the legs has been removed while the legs themselves are still kept inside the mask.

      Exception: if a bug has more than eight legs, or is very hairy, you don't have to exclude the background between the legs/hairs. However, you should still make sure that all parts of the bug (legs and hair included) are inside the mask (see Figure S1.5 for an example).

      iii. Mask should be relatively snug. Especially for small bugs or low-quality images, the edge of a bug can be blurred when zoomed in, creating a gradient between the bug (dark) and the background (light). When adjusting the mask, the edges of the mask should run approximately in the middle of such a gradient (see Figure S1.4).

Tip for facilitating adjustments: if the density of points along the mask is very high, it might be easier to make adjustments if you first reduce the density (see Figure S1.6).

iv. Mark entity as complete (shortcut: `c`).

We are annotating a large number of arthropods, and so each annotation should not take a lot of time. The rule of thumb is to spend no more than 10 seconds per bug mask once you are familiar with the tools and procedure. Use this as a guideline for how detailed you should be when adjusting the masks.

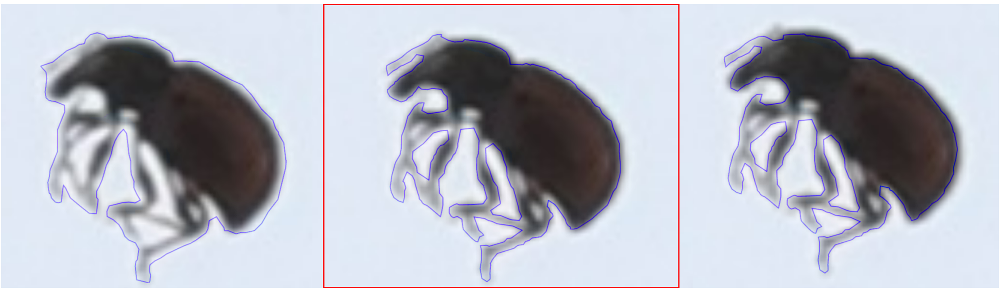

Figure S1.4. The edge of a bug can be blurred when zoomed in, creating a gradient between the bug (dark) and the background (light). When adjusting the mask, the edges of the mask should run approximately in the middle of such a gradient. Focusing on how the mask looks on the back of the insect in the picture: to the left, the mask is a bit too big, in the middle, it looks good, and to the right, the mask is a bit too small. The middle and right image also shows how areas of background between the legs should be removed.

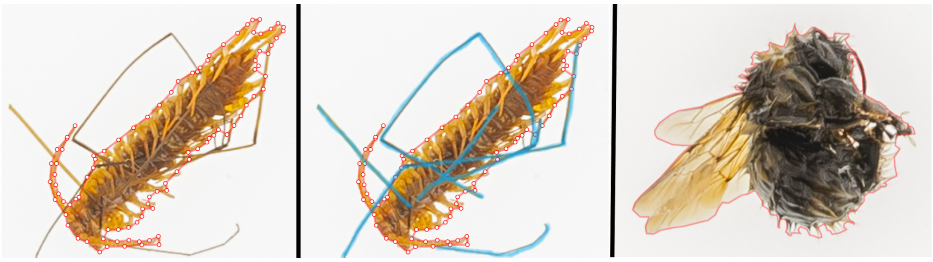

Figure S1.5. Example of acceptable masks for bugs that either have more than eight legs or are very hairy. All parts of the bug should be inside the mask, but not all areas of background between legs/chunks of hair must necessarily be removed. Which bugs are too hairy to make exact masks is a judgment call, but use the rule of 10 seconds per bug to guide you. The many-legged bug in this example is particularly tricky due to the debris tangled among the legs (marked with blue colour in the middle image).

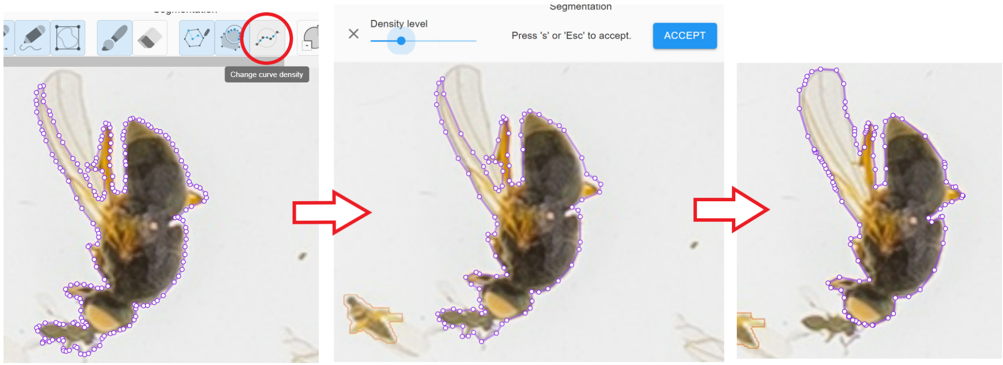

Figure S1.6. Reducing density of points along a mask. Use the tool "Change curve density", and click on two points to mark the section you wish to edit (click on the same point twice to edit point density along the entire mask). Slide the scale to the density you want. Press Esc to accept the changes. To the right, you can see the finalized mask.

c. If multiple bugs have been grouped together in the same mask, or if one bug is superimposed on another:

i. Select one bug to start with and adjust the mask for that bug, as described in 1b (see Figure S1.7).
ii. Add a new entity (shortcut: +). Rename it (shortcut: `n`) to ‘b’ (bug).
iii. Create a mask around the next bug, for example, using the bounding box tool.
iv. Adjust the mask as described in 1b.
v. Repeat steps ii-iv for each unmasked bug.
If you can’t distinguish which bug a certain body part belongs to (e.g. if the legs are tangled), leave the body part in question out of the mask.

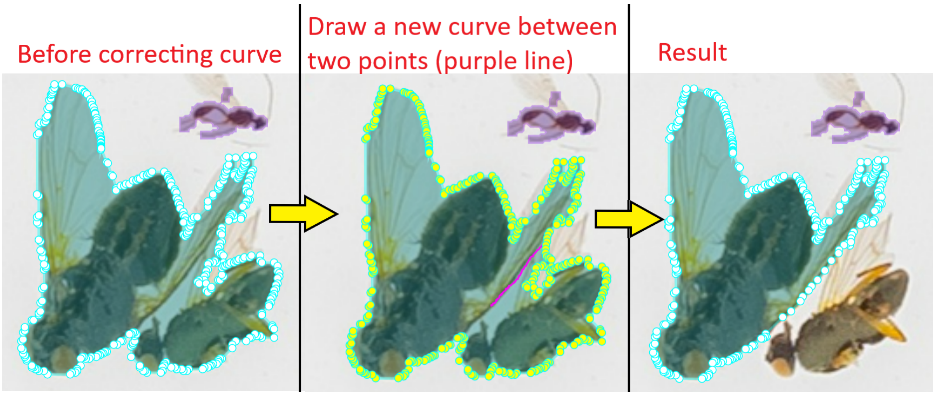

Figure S1.7. An example of two insects grouped within the same mask. Here, ‘Correct part of curve’ (shortcut: `s`) is used to draw a new edge to exclude one of the insects. The next step would be to create a new entity and draw a mask for the excluded insect.

d. For masks that are not bugs (i.e., “u”, “d”, or “e”), mark the mask as complete without making any adjustments.

2. Look through the image for arthropods missed by the watershed algorithm. For each unmasked bug that is fully contained in the image and not outlined with red colour (Figure S1.8):

a. Add a new entity and mask as described in 1c ii-iii.

b. Adjust the mask as described in 1b.

3. When you are done, mark all entities as complete, and click “Submit”.

S1.3.2 Tips and tricks

- Make use of the keyboard shortcuts in TORAS. They can be displayed by pressing “?” on your keyboard. Here are some examples:

Action	Keyboard Shortcut
Add entity	+
Paint	r
Erase	e
Rename entity	n
Create mask using bounding box	b
Complete entity	c

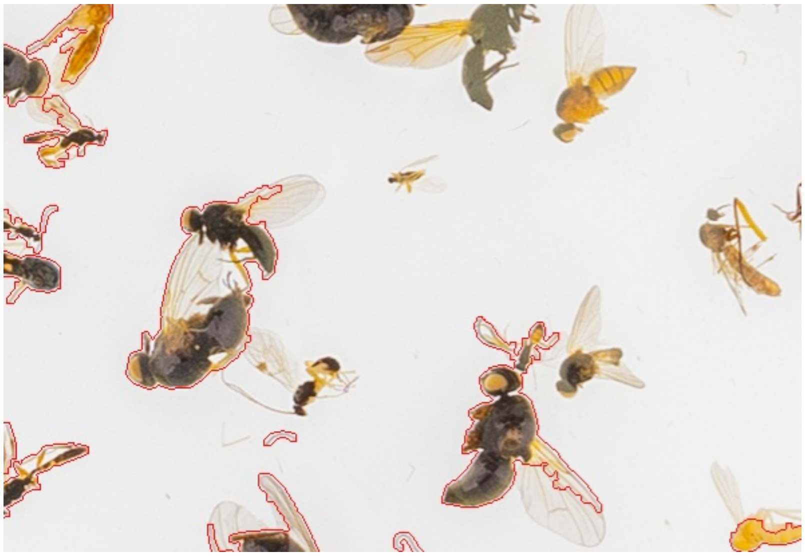

Figure S1.8. If the bugs are outlined with a red colour, it means that they are duplicated from another image, and you should not create a mask around them. The outlined bugs are found near the edges of the image.

- Sometimes when a new entity is created, TORAS zooms out to show the full image. This is optimized for the annotation of larger objects, but is not ideal when having to relocate a tiny bug in our images. To change this behaviour, click on options (right panel; 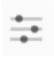) and make sure that 'Auto zoom on action' is turned off.

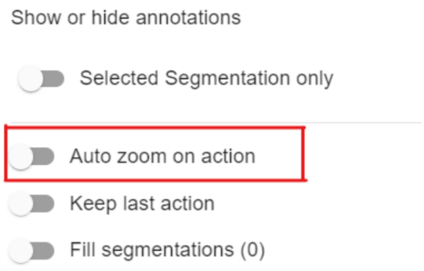

S1.4 Create labels (expert)

S1.4.1 The task

All bugs in the image should now have a refined segmentation mask, and the main task of the expert annotator is to assign a taxonomic label to each bug mask. As described previously, in addition to the images, we have data from DNA (meta-)barcoding of each sample; this sample-specific taxonomic information will be used to delimit the taxonomy from which you choose the labels, and might thus work as guidance when annotating the images.

DNA barcoding results in classification on varying taxonomic levels (mainly due to variable taxonomic cover of reference databases); some arthropods are identified to species, while others are only identified to order. It is also possible that specimens have been misidentified using the genetic data, for example, if two genera have overlapping genetic variation or if the reference database contains misidentified sequences. Therefore, you are free to choose taxonomic labels that are not part of the sample-specific suggested taxonomy (see instructions below).

NB: each original image is divided into sub-images before they are uploaded to TORAS (see previous description), and the sample-specific DNA data thus corresponds to multiple sub-images. Further, the original sample might have been split into two full images because of a large quantity of insects, in which case the number of corresponding sub-images is, of course, even higher.

Before labeling a bug, you are asked to validate the mask created by the non-expert annotator. This is mainly to catch any major mistakes, and you are not generally expected to adjust details of the mask (specific instructions are given below).

S1.4.2 Validate masks

For entities marked as ‘b’ (bug):

1. Check the mask of the non-expert annotator.
2. If it looks ok, go to classification.
3. If there are big mistakes (see examples below), first correct the mask (for how to do this, see below and consult the instructions above for the non-expert annotator), then go to classification.

   Examples of big mistakes:

   - a visual characteristic important for classification of the bug is not included in the mask (e.g., a wing, antennae)
   - a whole body part of the bug is not included in the mask
   - two bugs are grouped within the same mask
   - the mask includes a considerable amount of debris

To enable editing of the mask, first exit the classification pane by clicking anywhere outside of it. Then click on `Segmentation` of the entity you want to correct in the left pane:

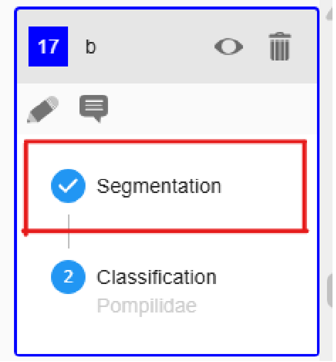

Then click on 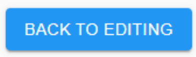

in the top right corner.

S1.4.3 Classification

When you choose an entity in the list on the left side of the screen, the classification window opens:

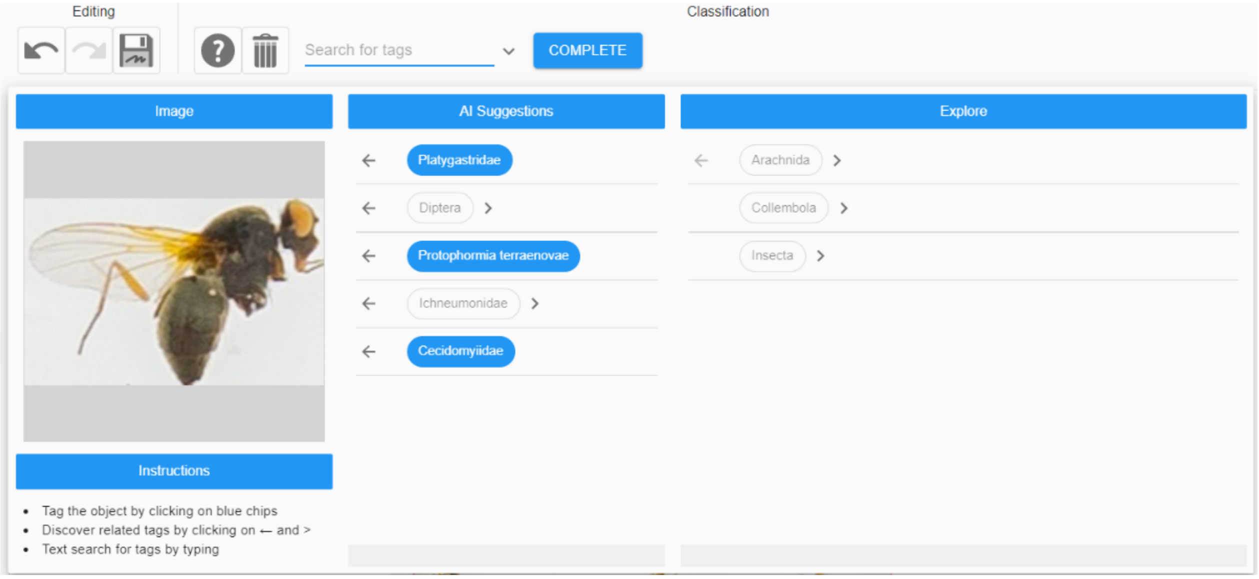

To the left, you see an image of the bug, and to the right, you can click through the sample-specific taxonomy to find an appropriate label. It is also possible to search for labels, using the search field above the classification window (‘Search for

tags’). *In the middle of the classification window, there are some AI-suggested labels – these are unlikely to be correct (the tool is meant to work for a wide variety of objects, and is not specialized on arthropods).*

If you can’t find the label you are looking for in the sample-specific taxonomy, you are free to add another label manually. You do this by writing the label in the search field and then pressing `ENTER`. We have prepared two Excel sheets containing all insect taxa in Sweden (where the majority of samples are from): one going down to family level (`dyntaxa_boldified_family.xlsx`) and one to species level (`dyntaxa_boldified_species.xlsx`). To avoid misspellings of manually added labels, please copy the taxonomic label from those files.

When you have chosen a label, press ‘Complete’ to mark the entity as done.

Now for the million-dollar question: **“How confident should I be of the classification?”** First of all, because of the large number of bugs to classify, you should not spend more than on average **10 seconds** on the classification of a single bug, so that puts some limitations on how detailed you can be. However, it will likely often be the image quality and lack of detail that limit the taxonomic level you can get to. In general, you should assign the most detailed taxonomic label you feel confident is correct. To capture more detailed information, it is also possible to give additional labels on a lower confidence level. There are two ways to do this:

1. When you want to choose **one** label: include a label on a higher taxonomic level. The label on the highest taxonomic level will then be interpreted as ‘high confidence’, while lower levels are interpreted as ‘low confidence’.

   Example: you think the bug you are looking at belongs to the family Ichneumonidae, but you could be mistaken. Therefore, you add both “`Ichneumonidae`” and “`Hymenoptera`” as labels. Hymenoptera will then be interpreted as ‘high confidence’, Ichneumonidae as ‘low confidence’.

2. When you want to choose **multiple** labels: choose multiple labels on any taxonomic level. The last common ancestor of all your chosen labels will be interpreted as ‘high confidence’, whether or not you include it as a label.

   Example:

   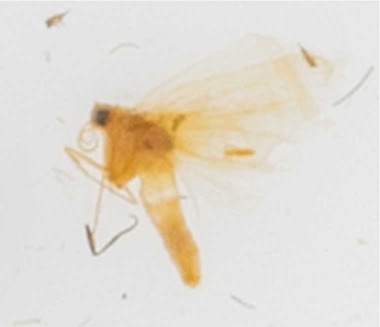

   You think this bug belongs either to Lepidoptera: Tortricidae or Lepidoptera: Geometridae. Therefore, you choose “`Tortricidae`”” and “`Geometridae`” as labels. They are both interpreted as ‘low confidence’ labels, but as they both belong to the order Lepidoptera, “`Lepidoptera`” is interpreted as a ‘high confidence’ label.

   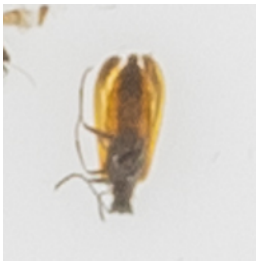

   Similarly, you think this is either a “`Coleoptera`” or “`Hemiptera`”, which are interpreted as ‘low confidence’ labels. They both belong to the class Insecta, so “`Insecta`” is interpreted as a ‘high confidence’ label.

As long as you give a single label to a bug, it will be interpreted as ‘high confidence’.

S1.4.4 Images of individual bugs

For each bulk image, we also have the full set of bugs photographed individually (the so-called “Keyence images”). These images can be used as guidance, for example, to get an idea of the abundance and morphological distribution of certain families. Images are grouped by order and family and can be accessed locally from an external hard drive.

S2 Supplementary methods

S2.1 Determining upsampling factor for tiles

If an entire bulk insect sample is downsampled to fit within a model's input size of 1024 × 1024 pixels, each insect is rendered at a lower resolution than in the original image, leading to blurred contours and fewer visible details – especially problematic for detecting small insects. An alternative is to divide the images into tiles to preserve visual details. Using smaller tiles than the required input size and instead upsampling the images to the target resolution can affect model performance. For example, presenting images to models at higher resolutions allows the model to spend more compute in processing the full input image, potentially improving its performance[1]. Correspondingly, we investigated how much the model's performance could be increased if the original images were upsampled before presenting them to the model.

To determine the optimal upsampling factor for our instance segmentation models, we performed training and inference while varying the dimensions of the bulk image tiles. As we decreased the size of our tiles, we needed to increase the upsampling rate to reach our fixed input size of 1024 × 1024 pixels. We performed this analysis on the validation set using the SAHI approach to ensure this hyperparameter selection was not based on the test partition. For each trial, we maintained a fixed input size of 1024 × 1024 pixels, a common input size for pretraining instance segmentation models[2–4]. Tiles smaller than this input size were upsampled to 1024 × 1024 pixels using bilinear interpolation. Thus, a tile size of 1024 × 1024 pixels would require a zoom factor of one to reach our desired input size, 512 × 512 pixels would require a zoom factor of two, and so forth until 128 × 128 pixels, which would require a zoom factor of eight.

As the zoom factor increases, the relative size of the arthropods in each tile increases, although each tile includes less spatial context, and more arthropods are cut between tiles. We observed that all three models achieve the best mask AP when they use 512 × 512 pixel tiles or a zoom factor of two (Figure S2.1), which we consequently used for all further experiments.

While increasing the zoom factor from one to two improves instance segmentation performance, higher zoom factors gradually degrade performance. Very small tiles, with zoom factors of six and eight, showed the worst mask AP across all models, suggesting that the increase in relative size is offset by the lack of spatial context when small tiles are used. Such context may be important for distinguishing small insects from surrounding debris. For example, as the tile size decreases, more insects are split between tiles. These partial insects may be more difficult to distinguish from debris, which includes loose insect legs and wings. This also complicates the inference stage as a) our models must correctly identify partial insects, and b) the SAHI algorithm must correctly merge fragmented insect predictions across tiles.

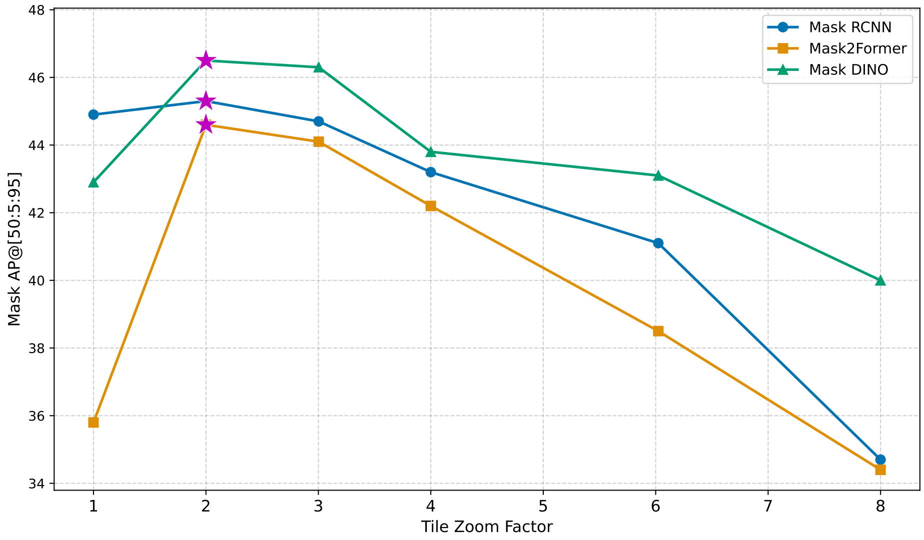

Figure S2.1. Validation mask AP versus tile zoom factor for our supervised baselines: Mask R-CNN, Mask2Former, and Mask DINO. For all three models, a zoom factor of two, corresponding to a tile size of 512 × 512 pixels, which is upsampled to a 1024 × 1024 pixels model input, gives the best instance segmentation performance.

S2.2 Implementation details for zero-shot methods

First, we assessed the Cut and Learn (CutLER) model[5], an unsupervised instance segmentation method trained on a dataset without human annotations. CutLER leverages a self-training process where the model is iteratively trained on its own

predictions to refine the quality of subsequent instance masks. For all CutLER experiments, we used a self-trained Cascade Mask R-CNN checkpoint (`cutler_cascade_final`)[5].

We then evaluated Grounding DINO[6,7] and Florence-2[8], which can localize objects of interest through text prompts. These text prompts can denote simple category names or referring expressions. For all Grounding DINO experiments, we provided the Grounding DINO-B model (`groundingdino_swinb_cogcoor`)[7] with the prompt `insect.`, where "." is used as a delimiter for different object classes. We then used the default box and text thresholds of 0.35 and 0.25, respectively.

For our Florence-2 evaluations, we used the publicly available `Florence-2-large-ft` checkpoint[8]. In addition to a text prompt, Florence-2 requires a task prompt denoting whether to perform captioning, detection, or other vision-language tasks.

Thus, we provided the following prompt to Florence-2: <OPEN_VOCABULARY_DETECTION> small brown-yellow insects. To suppress large bounding box predictions, we filtered out bounding boxes that occupy more than 40% of the area of a given 512×512 pixel tile (for comparison, the largest specimen in the dataset had a ground truth mask equal to 32% of a tile).

Lastly, we leveraged Gemini 2.0 Flash's spatial understanding capabilities to perform object detection[9]. With a temperature of 0.5, we provided the following system instructions: `Return bounding boxes as a JSON array with labels. Never return masks or code fencing. Limit to 50 objects. Never repeat or duplicate bounding boxes. If an object is present multiple times, return the same label for each instance.`

When performing detection, we used the following text prompt: `Detect the 2d bounding boxes of the small brown insects, ants, flies, and/or gnats. Exclude loose wings, legs, and debris.` As with Florence-2, bounding boxes occupying more than 40% of a tile were filtered out before being used as prompts for SAM 2.1. We performed inference with the `sam2.1_hiera_large` checkpoint without any fine-tuning on the MassID45 training set[10].

S2.3 Model evaluation with tailored confidence thresholds

To determine appropriate confidence thresholds for each model, we plotted precision-recall (PR) curves using their predictions on the MassID45 validation set (see Figure S2.2). We fixed the IoU threshold to 50%, indicating that we consider predicted masks as correct if they overlap by more than 50% in area with the ground truth. Each point corresponds to the precision and recall at a particular confidence threshold. Thus, for each model we selected the confidence threshold with the highest F1-score – the harmonic mean between precision and recall. This optimal confidence threshold is generally the point closest to the top-right corner of the PR curve, which represents perfect precision and perfect recall. These confidence thresholds can be interpreted as suggested operating points for each model when used on bulk images in a real-world setting.

Using these tuned confidence thresholds, we performed inference on the MassID45 test set, then filtered out any predictions below each model's confidence threshold. We then measured the number of TP, FP, and FN pixels predicted by each model on the test set (see Table S2.1). Consistent with our exemplar patch in Figure **??**, Mask DINO predicts the highest number of TP pixels and the lowest number of FN pixels, while Grounding DINO has the highest proportions of FPs and FNs. Mask R-CNN predicts the fewest FPs, while Mask2Former generally achieves a balance between Mask DINO and Mask R-CNN.

Table S2.1. Proportion of TP, FP, and FN pixels for each model on the MassID45 test set after tuning confidence thresholds.

Model	TP Area	FP Area	FN Area
Grounded SAM 2.1	712 478 (63.9%)	253 701 (22.8 %)	148 456 (13.3 %)
Mask2Former	783 319 (80.7%)	110 204 (11.4 %)	77 615 (7.99%)
Mask DINO	**787 067** (80.7%)	114 215 (11.7 %)	**73 867 (7.57%)**
Mask R-CNN	777 120 (81.4%)	**93 473 (9.79%)**	83 814 (8.78%)

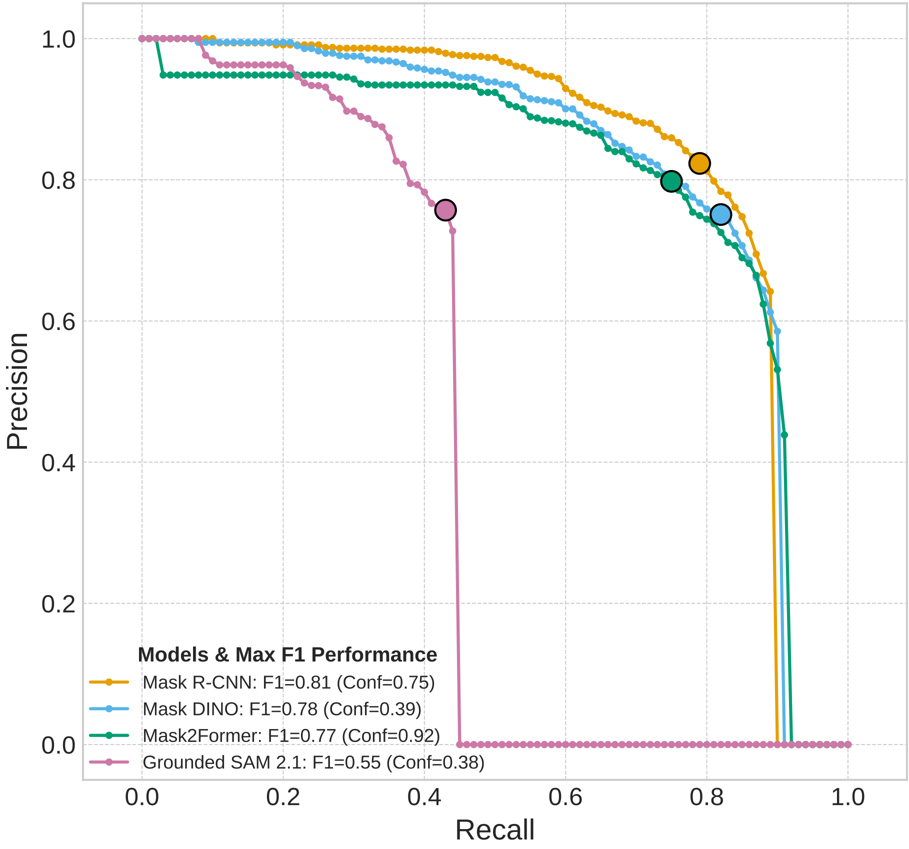

Figure S2.2. Precision-recall (PR) curves for the strongest zero-shot model (Grounded SAM 2.1) and the three supervised models (Mask R-CNN, Mask DINO, Mask2Former). We selected the confidence threshold for each model by finding the point on the PR curve with the highest F1-score.

References

1. Tan, M. & Le, Q. EfficientNet: Rethinking model scaling for convolutional neural networks. In Chaudhuri, K. & Salakhutdinov, R. (eds.) *Proceedings of the 36th International Conference on Machine Learning*, vol. 97 of *Proceedings of Machine Learning Research*, 6105–6114 (PMLR, 2019).
2. He, K., Gkioxari, G., Dollár, P. & Girshick, R. Mask R-CNN. In *2017 IEEE International Conference on Computer Vision (ICCV)*, 2980–2988, doi:10.1109/ICCV.2017.322 (2017).
3. Li, F. *et al.* Mask DINO: Towards a unified transformer-based framework for object detection and segmentation. In *2023 IEEE/CVF Conference on Computer Vision and Pattern Recognition (CVPR)*, 3041–3050, doi:10.1109/CVPR52729.2023.00297 (2023).
4. Cheng, B., Misra, I., Schwing, A. G., Kirillov, A. & Girdhar, R. Masked-attention mask transformer for universal image segmentation. In *2022 IEEE/CVF Conference on Computer Vision and Pattern Recognition (CVPR)*, 1280–1289, doi:10.1109/CVPR52688.2022.00135 (2022).
5. Wang, X., Girdhar, R., Yu, S. X. & Misra, I. Cut and learn for unsupervised object detection and instance segmentation. In *2023 IEEE/CVF Conference on Computer Vision and Pattern Recognition (CVPR)*, 3124–3134, doi:10.1109/CVPR52729.2023.00305 (2023).
6. Ren, T. *et al.* Grounded SAM: Assembling open-world models for diverse visual tasks. *arXiv preprint arXiv:2401.14159* doi:10.48550/arXiv.2401.14159 (2024).
7. Liu, S. *et al.* Grounding DINO: Marrying DINO with grounded pre-training for open-set object detection. In Leonardis, A. *et al.* (eds.) *Computer Vision – ECCV 2024*, 38–55 (Springer Nature Switzerland, Cham, 2025).
8. Xiao, B. *et al.* Florence-2: Advancing a unified representation for a variety of vision tasks. In *2024 IEEE/CVF Conference on Computer Vision and Pattern Recognition (CVPR)*, 4818–4829, doi:10.1109/CVPR52733.2024.00461 (2024).
9. Google DeepMind. Introducing Gemini 2.0: our new AI model for the agentic era. https://blog.google/technology/google-deepmind/google-gemini-ai-update-december-2024/#ceo-message/ (2024). [Online; accessed 23-April-2025].
10. Ravi, N. *et al.* SAM 2: Segment anything in images and videos. In *The Thirteenth International Conference on Learning Representations* (2025).
